# Inflammation mediated brain damage and cytokine expression in a maternally derived murine model for preterm hypoxic-ischemic encephalopathy

**DOI:** 10.3389/fsysb.2025.1517712

**Published:** 2025-07-01

**Authors:** Tyler C. Hillman, Braeden Jacobson, Kiara Piaggio Hurtado De Medoza, Marlene Lopez, Nicholas Iwakoshi, Christopher G. Wilson

**Affiliations:** ^1^ Lawrence D. Longo, MD Center for Perinatal Biology, Department of Basic Science, Loma Linda University, Loma Linda, CA, United States; ^2^ School of Medicine, Loma Linda University, Loma Linda, CA, United States; ^3^ Whittier College, Department of Biology, Whittier, CA, United States; ^4^ Department of Pediatrics, Loma Linda University Medical Center, Loma Linda, CA, United States

**Keywords:** hypoxia, ischemia, preterm, neonate, maternal inflammation, fetal inflammation, biomarkers

## Abstract

**Introduction:**

Preterm hypoxic-ischemic encephalopathy (pHIE) is a complex brain injury that contributes to chronic neural inflammation and neurological disorders. The signs and symptoms of in utero pHIE can often be overlooked, untreated or lumped into more generic conditions such as encephalopathy of prematurity (EOP). Clinical interventions like hypothermia and erythropoietin do not improve pHIE. We characterized a murine model for pHIE, which includes hypoxia and maternal factors as a cost-effective alternative to large animal models of HIE.

**Methods:**

We injected pregnant mouse dams with LPS to stimulate an inflammatory response on embryonic days 15–16 (E15–E16), and whole cage hypoxia exposures occurred from postnatal days 3 to 9. To quantify the development of inflammation in the pHIE model, we used immunohistochemistry to stain for Caspase-9 in the cortex (20 μm per slice) and then counted Caspase-9 positive cells using unbiased stereology. We stained brain tissue with MAP2 to quantify neuronal intermediate filament expression and staining using a machine-learning based image analysis approach. We quantified cytokines (IL-1β, IL-6, IL-10, IL-18 and TNF-α) using RT-qPCR and (IL-18) ELISA to characterize differential expression in all treatment groups. The pHIE animals were compared with controls (LPS-Normoxia, Saline-Hypoxia, Saline-Normoxia, and Naïve) and with a model of only hypoxia (10% O_2_) exposure in mouse pups.

**Results:**

The pHIE pups showed significantly higher expression of Caspase-9 throughout the cortex compared to Naïve pup brains (p < 0.05). MAP2 expression was significantly decreased (p < 0.05) between 1.5–6.0 mm of the brain compared to Saline-Hypoxia and Naïve animals. Both IL-1β and IL-10 expression in LPS-Hypoxia animals was significantly higher (p < 0.05) than in Saline-Hypoxia and Naive animals. TNF-α expression was not significantly different between LPS-Hypoxia and Saline-Hypoxia animals. However, both showed significantly different transcription, compared to Naive animals.

**Discussion:**

The model we describe here shows cortical damage similar to that seen in human HIE.

## 1 Introduction

Hypoxic-Ischemic encephalopathy (HIE) is caused by a lack of oxygen and blood flow to the developing brain, resulting in acute inflammatory upregulation that can lead to chronic inflammation and devastating long-term consequences. In developed countries, the rate of HIE is 1–8 in every 1,000 live births (Kurinczuk et al., 2010; Douglas-Escobar and Weiss, 2015; Namusoke et al., 2018; Ristovska et al., 2022). HIE-related mortality ranges from 15% to 20%; however, many infants suffer long-term morbidity such as developmental delay or cerebral palsy following the injury (Ristovska et al., 2022; Allen and Brandon, 2011). Furthermore, HIE can occur *in utero*, leading to preterm birth or death (Lawn et al., 2009; Trønnes et al., 2014). The reported rates of neonatal death resulting from HIE may be as high as 724,000 per year worldwide (Peebles et al., 2020). Clinically, intrauterine HIE diagnosis comes after the damage has already occurred. Historically, HIE and other CNS damage in premature neonates falls under the larger umbrella of encephalopathy of prematurity. Due to intrauterine injury, therapeutic agents are typically not administered in time to prevent or mitigate damage (Rice et al., 1981; Vannucci and Back, 2022) in these infants. A large body of HIE research relies on the Rice-Vannucci (RV) model, established in 1981, to investigate the mechanisms underlying HIE (Segovia et al., 2014; McCullough et al., 2017) and other hypoxic-ischemic brain injury. Despite its common usage, the RV model does not incorporate interactions between mother and fetus, which may be a key component to the initial insult if it occurs *in utero*.

**FIGURE 1:**
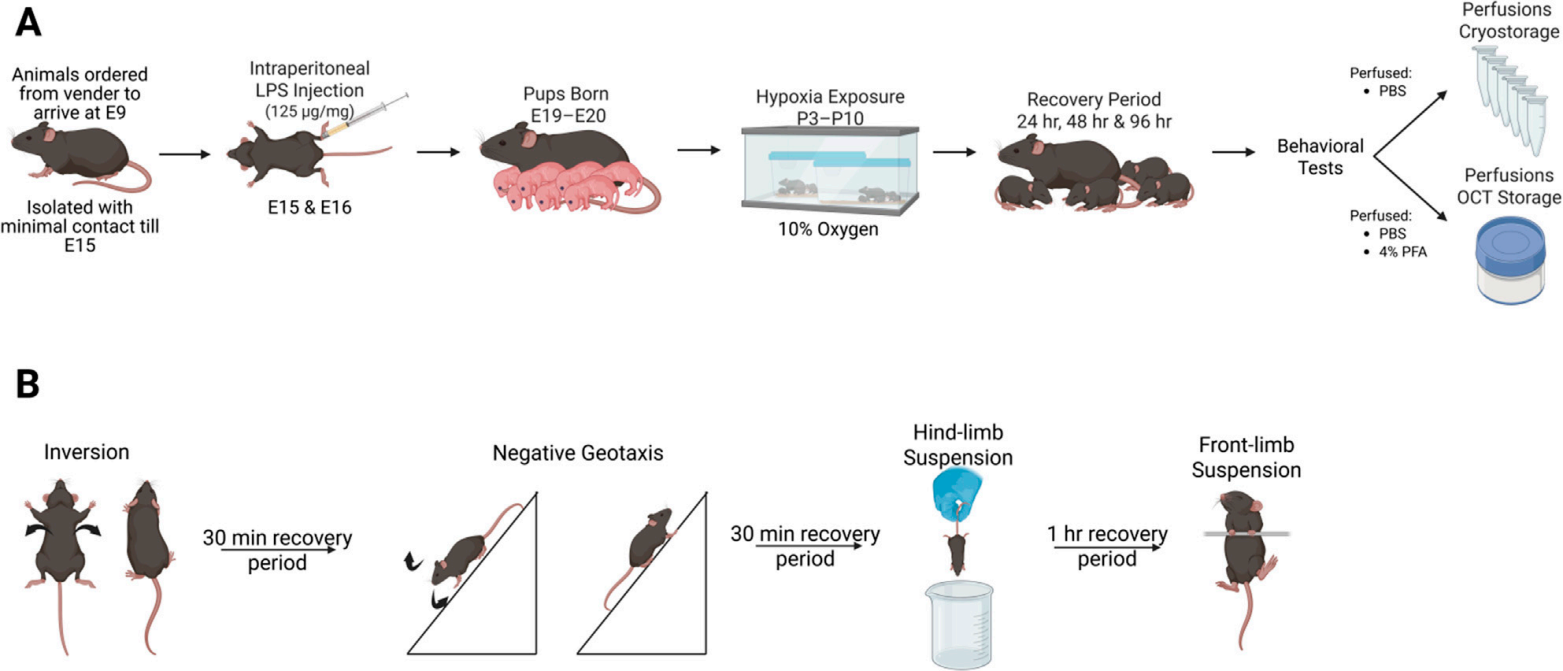
Diagram of experimental protocol for the LPS and Saline Hypoxia model **(A)**. The experimental protocol was originally modified from Lacielle et al. (2019). The experimental protocols for behavior are shown in **(B)**. Image created in BioRender. Hillman, T. (2025) https://BioRender.com/fv0lr5n

**FIGURE 2:**
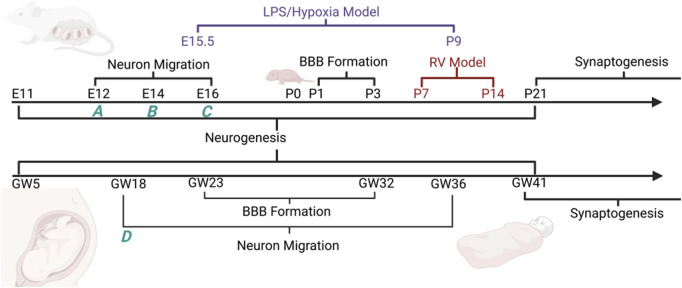
Diagram comparing the relative development of mouse vs. human during pregnancy. During neuron migration preplate formation is completed by E12 (A), neurons are migrating to cortical plate by E14 (B), and finally formation of mature cortical plate by E16 (C). In humans we see telencephalic to diencephalic migration occurring during the E18 to E36 time frame (D) (Semple et al. 2013). The LPS/Hypoxia model spans from E15.5 to P9 in contrast to the RV model which spans from P7 to P14. “Created in BioRender. Hillman et al. (2022) BioRender.com/c20e830.

We set out to characterize a maternal-derived murine model for HIE which includes maternal factors that may influence neonatal outcome (Segovia et al., 2014; McCullough et al., 2017; Chen et al., 2021). Current research points out connections between maternal and fetal communication in perinatal injury and an increase in autism (Teo et al., 2017; Lacaille et al., 2019). Additional studies focused on maternal obesity, low-level inflammation, and fetal neural inflammation post-birth, show that maternal inflammation can lead to alterations in CNS inflammatory state in the fetus (Matta et al., 2019; Semple et al., 2013). When considering the pathophysiological substrate for pHIE, the impact of maternal factors on fetal outcome have often been ignored in animal models. We used a modified version of a model originally developed by Lacaille and colleagues (Figure 1), to address the role of maternal stress and inflammation as a contributor to pHIE. We quantified the expression of apoptotic and inflammatory markers, in addition to other markers of brain injury in the developing cortex of mice (Lacaille et al., 2019). This model allows for initiation of maternal inflammation during early fetal neural development and hypoxia exposure during the developmental window for glia cell differentiation (Figure 2) (Semple et al., 2013).

Following exposure to hypoxia, cells can suffer metabolic failure resulting from mitochondrial dysfunction and failure of ATP synthesis (Arteaga et al., 2017; Behringer et al., 2009; Li et al., 2017; Nakajima et al., 2000). These cells then undergo necrosis and release damage-associated patterns (DAMPS) locally (Murao et al., 2021). Microglia with toll-like receptor 4 (TLR-4) are activated by these DAMPs, shifting the microglia into a pro-inflammatory state and stimulating the expression and release of cytokines (Li et al., 2017; Nakajima et al., 2000). After the initial cytokine release, microglia are activated through IL-1R and shifted to a pro-inflammatory state (Douglas-Escobar and Weiss, 2015; Li et al., 2017; Kaushik et al., 2013; McAdams and Juul, 2012; Ziemka-Nalecz et al., 2017). Additionally, these cytokines affect the developing blood-brain barrier (BBB) by increasing its permeability (Capaldo and Nusrat, 2009; Pun et al., 2009; Takeshita and Ransohoff, 2012). A key challenge has been finding a rodent model that shows similar expression of cytokines and cellular damage as that seen in the clinical presentation of HIE.

Prior work, informed by both animal and clinical studies of HIE, has shown that damage from cytokine expression and downstream CNS damage are key, defining features of HIE. These studies drive home the need for a clearer understanding of the role of neuroinflammation and a more realistic model of preterm HIE. Expression of IL-1β (Yoon et al., 1997; Nelson et al., 1998; Oygür et al., 1998; Aly et al., 2006; Orrock et al., 2016; Massaro et al., 2018; Perrone et al., 2018), IL-6 (Yoon et al., 1997; Nelson et al., 1998; Aly et al., 2006; Orrock et al., 2016; Shalak et al., 2002; Chiesa et al., 2003; Jenkins et al., 2012), IL-10 (Orrock et al., 2016; Massaro et al., 2018; Perrone et al., 2018; Jenkins et al., 2012) and IL-18 (Perrone et al., 2018; Minagawa et al., 2002; Šumanović-Glamuzina et al., 2017) have been shown to be significantly increased compared to healthy newborns.

Activation of the TLR2/4 pathways leads to neuroinflammation and downstream expression of both pro- and anti-inflammatory cytokines. Initial damage occurs with cell death and release of DAMPS (damage-associated molecular patterns), which then bind to TLR2/4 and cause activation of the MYD88/MapK/NF-κB pathway (Li et al., 2017; Nair et al., 2012; Khatib et al., 2019). Once present, IL-1β can activate IL-1R on surrounding cells leading to further activation of the MYD88/MapK/NF-κB pathway and release of additional cytokines (Li et al., 2017; Nair et al., 2012; Khatib et al., 2019). Furthermore, IL-1R activation leads to increased expression of Caspase-9 and creation of the apoptosome in the cell (He et al., 2022). Clinically, these cytokines are important because preterm infants show prolonged expression of pro-inflammatory cytokines even when treated with current therapies like hypothermia and erythropoietin (Douglas-Escobar and Weiss, 2015; Nair et al., 2012; Parikh and Juul, 2018; Cainelli et al., 2021; Edmonds et al., 2021). As a result, outcomes for preterm infants are worse than their full-term counterparts (Arteaga et al., 2017; Parikh and Juul, 2018; Lv et al., 2015). Recent work has shown that the RV model is deficient in representing clinical HIE; providing impetus for a model that interrogates the role maternal factors play in the inflammatory pathway leading to HIE in preterm infants. Clinically this is important since most targeted therapies of HIE must be administered within 6 h of the onset or detection and are not effective or contra-indicated in pHIE patients (Shalak et al., 2002; Bustelo et al., 2020; Faix et al., 2025). Despite this knowledge, treating preterm infants within that narrow time window may be impossible due to the difficulty of determining the exact time of the events initiating hypoxic-ischemic injury.

The goal of this study was to quantify inflammatory markers, injury severity, and early behavioral improvement in a new model of pHIE that incorporates both maternally derived factors and hypoxic injury to the neonate. We hypothesized that a pHIE model incorporating maternal stress more closely resembles the patterns of injury associated with human HIE. To test this hypothesis, we assessed perinatal brain injury and inflammation using mRNA and protein quantification, quantification of Caspase −9, MAP2, and behavior in mice. We focused on a window of 48–96 h after birth. We quantified both pro-inflammatory cytokine (IL-1β and TNF-ɑ) and anti-inflammatory cytokine (IL-6 and IL-10) message and protein. We also quantified Caspase-9 as a marker of inflammation-initiated cell death along with MAP2 localization in regions associated with neuronal damage. MAP2 is a neuronal microtubule protein that is decreased following ischemic stroke and neuronal damage (Guo et al., 2021). Additional supporting data included behavior, morphology, and physiology.

## 2 Methods

### 2.1 Study approval

All animal procedures were approved by Loma Linda University Institutional Animal Care and Use Committee (IACUC 18-044 and IACUC 22-015). All animal work was conducted in the LLU Animal Care Facility (ACF) in a light-cycle and temperature controlled environment.

### 2.2 Animal model

Our protocol was based on that used by Lacaille and colleagues (Lacaille et al., 2019). Briefly, timed pregnant C57Bl6J dams (Charles-River, Cat. No. 00027) were ordered at embryonic age 8 (E8). Animals were housed in pairs and isolated within our animal care facility to minimize environmental stress. The use of privacy film limited the dams’ exposure to external visual stimuli. On E15.5 and E16.5, we injected the dams with 125 ng/kg of LPS solution. Dams were allowed to recover and give birth naturally. On postnatal day three (P3), the dams and her pups were introduced to hypoxia (exposure from P3 to P9 at 10% oxygen), by using a nitrogen generator and purpose-built hypoxia chamber (Hillman et al., 2022) with an extensive array of sensors to detect oxygen, pressure, temperature, humidity, and volatile organic compounds. Following hypoxia exposure, the dams and pups were allowed to recover a normal atmosphere, and tissue (heart, brain, lung, and kidney) was harvested at 24 h (P10), 48 h (P11), and 96 h (P13) post hypoxia exposure (Figure 1).

### 2.3 Tissue collection

We collected tissue for protein and RNA analysis after transcardial reperfusion with 5 mL 1x PBS at a rate of 2 mL/min (Peristaltic pump, Harvard Apparatus). During reperfusion pups were anesthetized using isoflurane at 3% induction and 2% during the surgery, 100% oxygen with a flowrate of 1.0 L/min. The brain, heart, lung, tail clip, and spleen were removed and snap-frozen using liquid nitrogen. All samples were stored at −80°C until use in experiments.

Tissue for immunohistochemistry was perfused via transcardial perfusion with 5 mL PBS at a rate of 2 mL/min, followed by perfusion with 5 mL of 4% PFA (2 mL/min) (Peristaltic pump, Harvard Apparatus). During reperfusion pups were anesthetized using isoflurane at 3% induction and 2% during the surgery, 100% oxygen with a flowrate of 1.0 L/min. The spleen and tail clip were removed before perfusion and frozen using liquid nitrogen. The brain, lung, and heart were isolated following perfusion and stored in 4% PFA for an additional 24–48 h, followed by incubation in 30% sucrose solution for 24–72 h.

### 2.4 Behavioral testing

All behavioral tests were recorded using the Surfola action camera (SF430 and SF530) at a resolution of 4K, 60 FPS. Videos were edited and analyzed using Kdenlive (Mardelle et al., 2024) and indexed by minute, second, and frame number. The frame number was converted to fractional seconds by dividing the total number of frames by 60 FPS to yield time. All behavioral tests were based on Feather-Schussler and Ferguson (Feather-Schussler and Ferguson, 2016) and included: inversion, negative geotaxis, front-limb hang, hindlimb hang. For details about these tests, please refer to their published paper and the Supplementary Document.

### 2.5 Tissue sectioning

Following perfusion, brains were embedded in OCT (Electron Microscopy Sciences, 62500-01) and stored at −80°C until cryos-sectioned (Leica Cryostat) at 20 μm thickness. Sections were taken from the first appearance of the cortex starting at the caudal end of the olfactory bulb based on the Allen brain matrix (starting slice similar to image 22 of the mouse Coronal atlas (Allen Institute for Brain Science, 2004).

For Caspase-9 staining, slides were removed from the −20°C and kept at room temperature for 10 min. We placed slides on a 40°C plate warmer to dry for 12 h. Slides were washed three times in 1x PBS at room temperature for 10 min. To block peroxidase, slides were incubated in 1% H_2_O_2_ (Sigma-Aldrich H1009) for 15 min at room temperature. We blocked samples in 200 μL of blocking solution (5% NGS (Sigma-Aldrich, G9023), 1% BSA (Life Technologies, 37525), 0.2% Triton in PBS) for 2 h with coverwells (Electron Microscopy Sciences, 70327–05) to prevent evaporation. Excess solution was removed and we added 200 μL of primary antibody solution (2% NGS (Sigma-Aldrich, G9023), 1% BSA (Life Technologies, 37525), 0.1% Triton, 1:1000 Anti-Caspase-9 antibody (AB52298) or 1:1000 Anti-MAP2 antibody (AB183830) in PBS) for 2 h at room temperature or overnight at 4°C with coverwells. Slides were washed then incubated in 200 μL secondary antibody solution (2% NGS (Sigma-Aldrich, G9023), 1% BSA (Life Technologies, 37525), 0.1% Triton, 1:1000 secondary antibody (AB205718) in PBS) for 2 h at room temperature with coverwells. We washed the slides then added 200 μL of DAB substrate (AB64238) onto each slide and allowed it to incubate at room temperature for 10 min. We then submerged the slides in Hematoxylin (AB245880) for 1 min followed by 3 washes in 1× PBS for 5 min each wash. Slides were dried and 125 μL of paramount was used to seal coverslips.

### 2.6 Cell counting

We counted caspase-9 positive cells using *Sterologer* (SRC Biosciences, Tampa, FL) to obtain an unbiased count of stained cells. The cortex of the brain was outlined at 4× magnification and verified using the Allen Brain Atlas (Allen Institute for Brain Science, 2004). We selected reference markers and counted cells at 40× magnification. Cell counts were recorded for counts with area and count CEs (coefficient of error) below 0.15 for each section. Counts were normalized to total volume (µm^3^) and divided by thickness (µm) to get counts per area (Counts/µm^2^) (Siebold et al., 2018).

For MAP2 area calculations, Visiopharm (Visiopharm, Denmark 2024) was utilized to quantify the area of MAP2 stain, hematoxylin counterstain, and tissue total area. Utilizing Visiopharm (Visiopharm, App 10016, version 2023.01.2.13695), the total area of MAP2 and Caspase-9 were quantified (for specific settings, please see Supplementary Material). We used Shapiro-Wilks to test for the normality of the data (R, dplyr). Due to the non-normal distribution of data, we used nonparametric statistical analysis (R, rstats) methods. We used Dunn and Kruskal–Wallis tests to compare between groups.

### 2.7 RNA isolation

RNA extraction was performed using the miRNAeasy Mini Kit (Qiagen 217084). We followed standard miRNAeasy Kit instructions with minor adjustments. After adding QIAzol (Step 3) we left the samples at room temperature for 3 min then frozen at −80°C for less than 1 month. Later, we thawed samples at 36°C for 5 min and allowed them to rest at room temperature for an additional 3 min (per Qiagen protocol). Finally, we transferred the spin column into a new 1.5 mL tube, added 50 µL of RNase-free water, and centrifuged at 8,000× G for 1 min at room temperature to extract RNA. We isolated 8 µL of each sample for Nanodrop quantification. Sample concentration (ng/µL), as well as A260/A280 and A260/A230, were recorded and assessed for sample purity.

### 2.8 RT-qPCR

Extracted RNA was reverse transcribed into cDNA using the RevertAid First Strand cDNA Synthesis Kit (Thermo Fisher Scientific, K1621). To create the DNA solution (12 µL), are added and mixed: 2 µL tRNA (0.5 ng/μL starting concentration), 1 µL primer (Random Hexamer, BioRad), and the remainder with molecular grade water (VWR, VWRL0201-0500). The protocol was optimized for 1.0 ng starting total RNA. The Master Mix was created with the following: 5x reaction buffer, 1 µL RiboLock RNase Inhibitor, 2 µL 10 mM dNTP Mix, and 1 µL RevertAid M-MuLV RT. DNA Solution and Master Mix were gently mixed. Amplification conditions were 25°C (5 min), 42°C (60 min), and 70°C (5 min). Samples were then diluted with 20 µL RNase-free water. The primers used for real-time PCR were GFAP (positive control), Actin (house-keeping gene), IL-1β, IL-6, IL-10, IL-18 and TNF-ɑ (Supplementary Table S1). For quantitative PCR (qPCR), cDNA was amplified using SYBR Green Master Mix (Applied Biosystems, 4368577) on a CTX-96 fluorescent thermocycler (BioRad, CTX-96).

### 2.9 Protein isolation/quantification

Samples were isolated using N-PER reagent (Thermo Fisher Scientific, 87792) with a final concentration of 1× HALT and 1× EDTA protease inhibitors (Thermo Fisher Scientific, PI78429). Samples were homogenized at a ratio of 1 g tissue to 10 mL N-PER reagent on ice for 20 min. After the sample was incubated, the supernatant was isolated by spinning at 10,000 × G for 10 min at 4°C to pellet the cell debris. The supernatant was isolated and total protein was quantified using a BCA kit (Thermo Fisher Scientific, 23235). Samples were diluted to 4.0 mg/mL concentration using N-PER reagent. When running ELISAs for protein quantification (IL-18 (AB216165), IL-1β (AB197742) samples were diluted with reagent diluent provided by kits at a ratio of 1:12 for IL-18 and 1:2 for IL-1β.

### 2.10 Statistical analysis

For all experiments, statistical analysis was performed using nonparametric analysis after checking data for normality (Shapiro-Wilks, R v 4.3.2). Three different methods were used to analyze the data. Differences between the animal ages of each group (e.g., LH at P10, P11, and P13), comparisons between different groups at each age (e.g., LH, SH, Naïve, at P11), and post-insult times (e.g., 24, 48, and 96 h post-injury). For each of these methods, we used the Kruskal–Wallis with Dunn pairwise test (R v 4.3.2 with ggplot2 v 3.4.4, ggstatsplot v 0.12.2, tidyr v 1.3.1, ggpubr v 0.6.0, and FSA v 0.9.5, Supplementary Tables S6, S7) to determine significance (p-value threshold of 0.05, Supplementary Table S6). For caspase-9 brain section data a Welch test with Games-Howell pairwise was used for statistical comparison (Games-Howell, R v 4.3.2). For physiology data, additional analysis was performed using linear regression models (R v 4.3.2). Experimental n values can be found in Supplementary Tables S2, S3 and all values in results were reported as mean ± standard deviation and are available in Supplementary Tables S4, S5.

## 3 Results

### 3.1 Morphology

**FIGURE 3:**
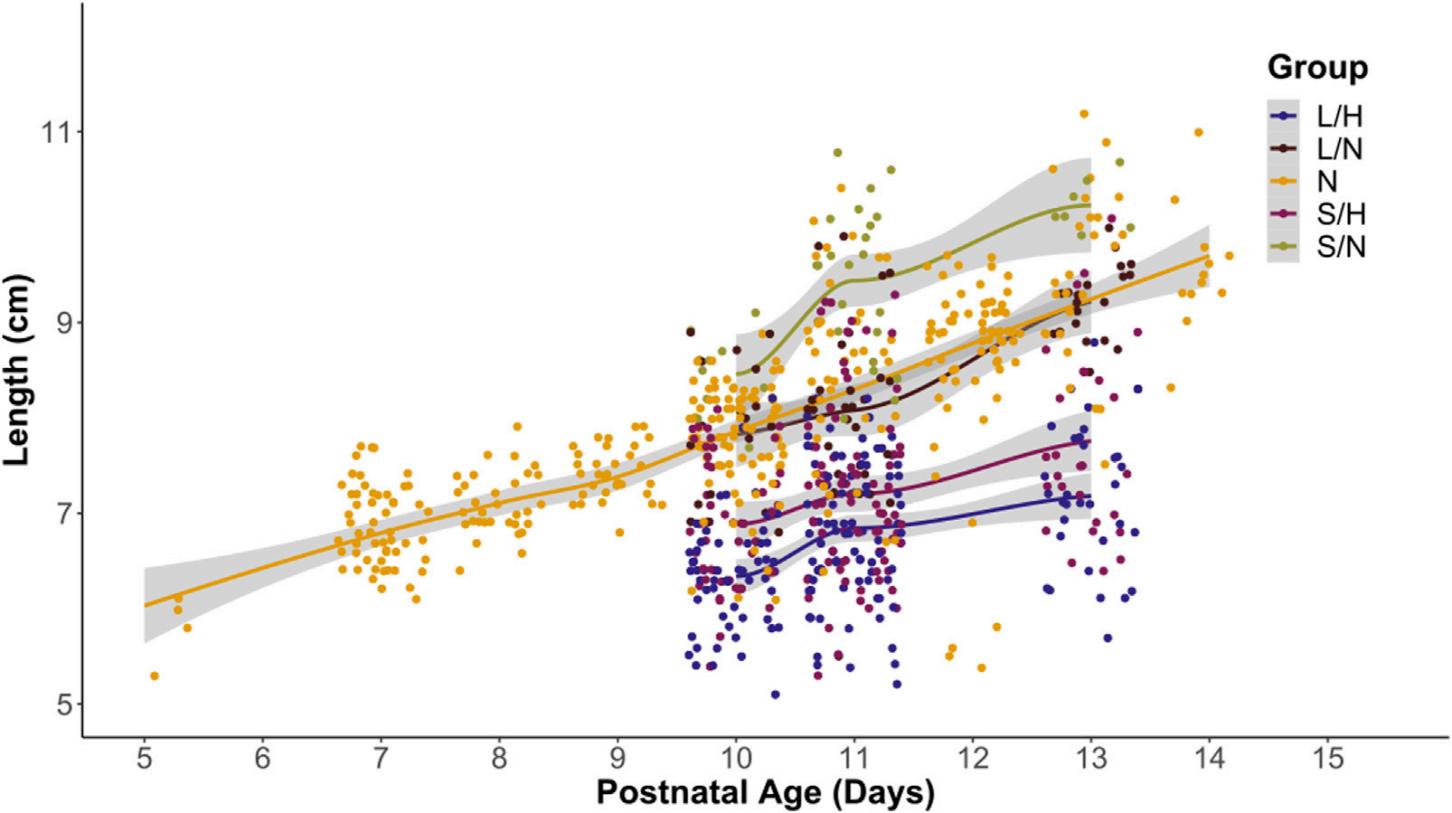
Length of pups vs. postnatal ages. Most groups cluster around the Naïve (orange) length. However, LPS/Hypoxia (LH) and Saline/Hypoxia (SH) are shorter than the other animals at a similar age. The offset of the P10 through P13 pups is similar to Naïve at P7.5 to P10. Linear regression analysis showed length and gestational age was significantly different with a p-value of 2.2 × 10^−16^, with each group having a p-value of 1.0 × 10^−10^ or lower.

**FIGURE 4:**
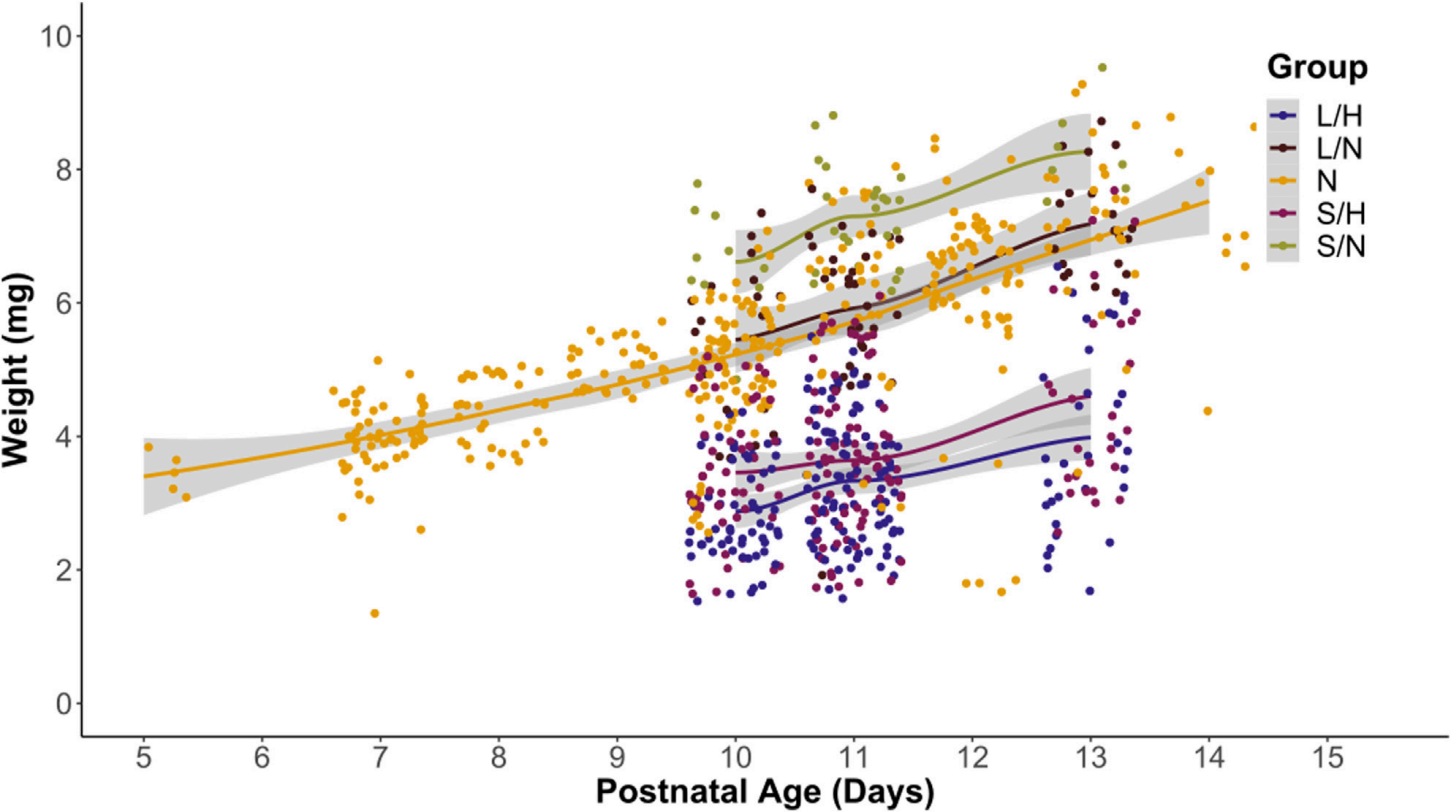
Pup weight vs. postnatal age. Most cluster around the Naïve group. LPS/Hypoxia (LH) and Saline/Hypoxia (SH) are lower than other groups across postnatal age. P10 through P13 pups are similar to Naïve animals at P5 to P8. Linear regression showed significant differences (p-value of 2.2 × 10^−16^, with each group p-value of 2.3 × 10^−7^ or lower).

We recorded length and weight of pups in all groups. Based on clinical data, babies with pHIE show growth restriction and low APGAR scores (Douglas-Escobar and Weiss, 2015; Schmidt and Walsh, 2010). We used linear regression to determine if there was no significant relationship between the length and group (Figure 3) and weight and group (Figure 4). We found a significant relationship between animal length and surgical and animal weight and surgical group (p-value = 2.2 × 10^−16^). Across groups, (Figure 3), the LPS/Hypoxia (p-value <1 × 10^−10^) animals have a shorter length. Similarly, group and weight (Figure 4) had a significant multivariable linear regression model (p-value = 2.3 × 10^−7^) for all groups. Saline/Hypoxia animals also had reduced size and weight compared to the other groups and were larger than the LPS/Hypoxia animals. This shows that hypoxia alone impacts pup growth. However, maternal inflammation further reduced the size of the pups compared to the Saline controls.

### 3.2 Behavior

#### 3.2.1 Negative geotaxis

All groups showed a consistent trend in ability to turn uphill within 10 s of release. There are changes with age for each group with the Naïve group having the most notable change over the 5 days tested. Kruskal–Wallis with Dunn Pairwise analysis showed a significant change between time points in the Naïve group, there was a significant decrease in time to turn uphill between P11 and P13 (9.7 ± 5.7 vs. 7.4 ± 7.3, p-value = 0.048) (Supplementary Figure S1). When comparing the difference between groups at different ages, at P10 there is a significant difference between LPS/Hypoxia and Saline/Hypoxia (8.3 ± 4.6 vs. 7.3 ± 6.9, p-value = 1.07 × 10^−2^) animals. At P10, Saline/Hypoxia has a significant decrease when compared to N (7.3 ± 6.9 vs. 11.1 ± 7.0, p-value = 4.16 × 10^−5^) (Kruskal–Wallis with Dunn pairwise test).The Saline/Hypoixa animals were also statistically different from Naïve at P11 (7.1 ± 6.9 vs. 9.7 ± 5.7, p-value = 1.31 × 10^−3^) group, with Saline/Hypoxia having a quicker flip time (Kruskal–Wallis with Dunn pairwise test) (Supplementary Figure S1). The final difference was seen at P13 where the LPS/Hypoxia group had a statically longer time to turn around than the Naïve (9.9 ± 6.4 vs. 7.4 ± 7.3, p-value = 8.21 × 10^−5^) and Saline/Hypoxia (9.9 ± 6.4 vs. 5.5 ± 4.1, p-value = 0.01) animals (Kruskal–Wallis with Dunn pairwise test) (Supplementary Figure S2).

#### 3.2.2 Inversion

The inversion test showed shorter flip times with increasing age. There were significant differences in flip time with increased ages of the LPS/Hypoxia, Naive, and Saline/Hypoxia animals (Kruskal–Wallis with Dunn pairwise test). The LPS/Hypoxia group showed a significant difference between P10 and P11 (2.2 ± 4.5 vs. 1.6 ± 1.6, p-value = 1.22 × 10^−6^) and P11 and P13 (1.6 ± 1.6 vs. 1.1 ± 0.7, p-value = 1.55 × 10^−3^). The Naïve group showed significant differences across: P8 and P9 (2.3 ± 1.7 vs. 1.2 ± 0.6, p-value = 4.55 × 10^−8^), P9 and P10 (1.2 ± 0.6 vs. 3.6 ± 6.8, p-value = 5.47 × 10^−4^), P10 and P11 (3.6 ± 6.8 vs. 1.8 ± 2.1, p-value = 2.05 × 10^−3^). Finally, there were significant differences when comparing the Saline/Hypoxia group at ages P10 and P11 (2.2 ± 4.5 vs. 1.6 ± 1.6, p-value = 3.55 × 10^−3^) and P11 to P13 (1.6 ± 1.6 vs. 1.1 ± 0.7, p-value = 2.63 × 10^−3^) (Supplementary Figure S3). We did not observe differences in general trends between the groups the improvement of flip time correlates with increased animal age (Supplementary Figure S4). We also wanted to look at the differences between the groups at different ages. At P10 we observed significant differences between the Saline/Hypoxia and LPS/Hypoxia (1.4 ± 1.1 vs. 2.2 ± 4.5, p-value = 4.15 × 10^−5^) and Naive (1.4 ± 1.1 vs. 3.6 ± 6.8, p-value = 3.82 × 10^−5^) groups. At P11 we observed differences between: LPS/Hypoxia and Saline/Hypoxia (1.6 ± 1.6 vs. 2.2 ± 2.8, p-value = 1.40 × 10^−3^), Naïve and Saline/Hypoxia (1.8 ± 2.1 vs. 2.2 ± 2.8, p-value = 1.81 × 10^−3^), (Supplementary Figure S3).

#### 3.2.3 Forelimb hang

**FIGURE 5:**
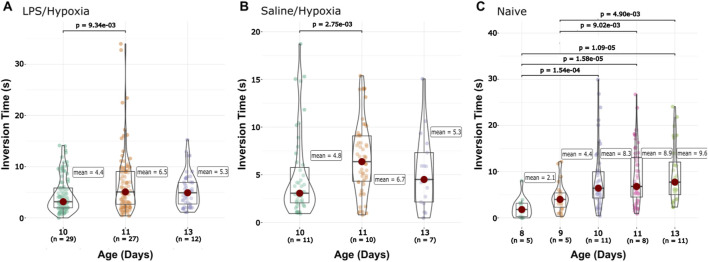
Front-limb Hang data. The Saline/Hypoxia **(B)** groups show no significant changes between time points. The LPS/Hypoxia **(A)** group showed significant differences at P10 and P11 **(C)**. Naïve animals showed a difference between P9 and P10, and between P8 and P11/P13, and P9 and P11/P13.

For the forelimb hang, we see that the Naïve group displayed a gradual increase in the time the animals were able to stay suspended by their front legs with age (Supplementary Figure S6). However, no other groups share this trend. The LPS/Hypoxia and Saline/Hypoxia groups both showed flat trends over the 96 h (Supplementary Figure S5). Both the Saline/Hypoxia and LPS/Hypoxia groups fell below the Naïve and flatten around P13 (Supplementary Figure S6A). We compared the intragroup differences between the animals’ ages, LPS/Hypoxia significantly increased between the P10 and P11 (4.4 ± 3.4 vs. 6.5 ± 5.8, p-value = 9.34 × 10^−3^) but no change between P11 and P13 (5.33 ± 3.2). The Naïve group had no significantly difference between age ranges: P8 (2.1 ± 2.1) to P9 (4.4 ± 3.0), P9 (4.4 ± 3.0) to P10 (8.3 ± 6.8), P10 (8.3 ± 6.8) to P11 (8.9 ± 6.2), and P11 (8.9 ± 6.2) to P13 (9.6 ± 6.3) did not show any significant change between ages (Figure 5). The Saline/Hypoxia group also showed a significant increase from P10 to P11 (4.8 ± 4.4 vs. 6.7 ± 3.7, p-value = 2.82 × 10^−3^) but no change from P11 to P13. At P10 there was a significantly longer time for Naïve compared to Saline/Hypoxia (8.3 ± 6.8 vs. 4.8 ± 4.4, p-value = 2.07 × 10^−3^) and LPS/Hypoxia (8.3 ± 6.8 vs. 4.4 ± 3.4, p-value = 2.20 × 10^−4^) group at P10. At P11 there was no difference across all groups. Lastly, at P11 and P13 there was a significantly higher time for Naïve when compared to LPS/Hypoxia (P13, 9.6 ± 6.3 vs. 5.3 ± 3.2, p-value = 2.02 × 10^−2^; P11, 8.9 ± 6.2 vs. 6.5 ± 5.8, p-value = 1.73 × 10^−2^).

#### 3.2.4 Hindlimb hang

**FIGURE 6:**
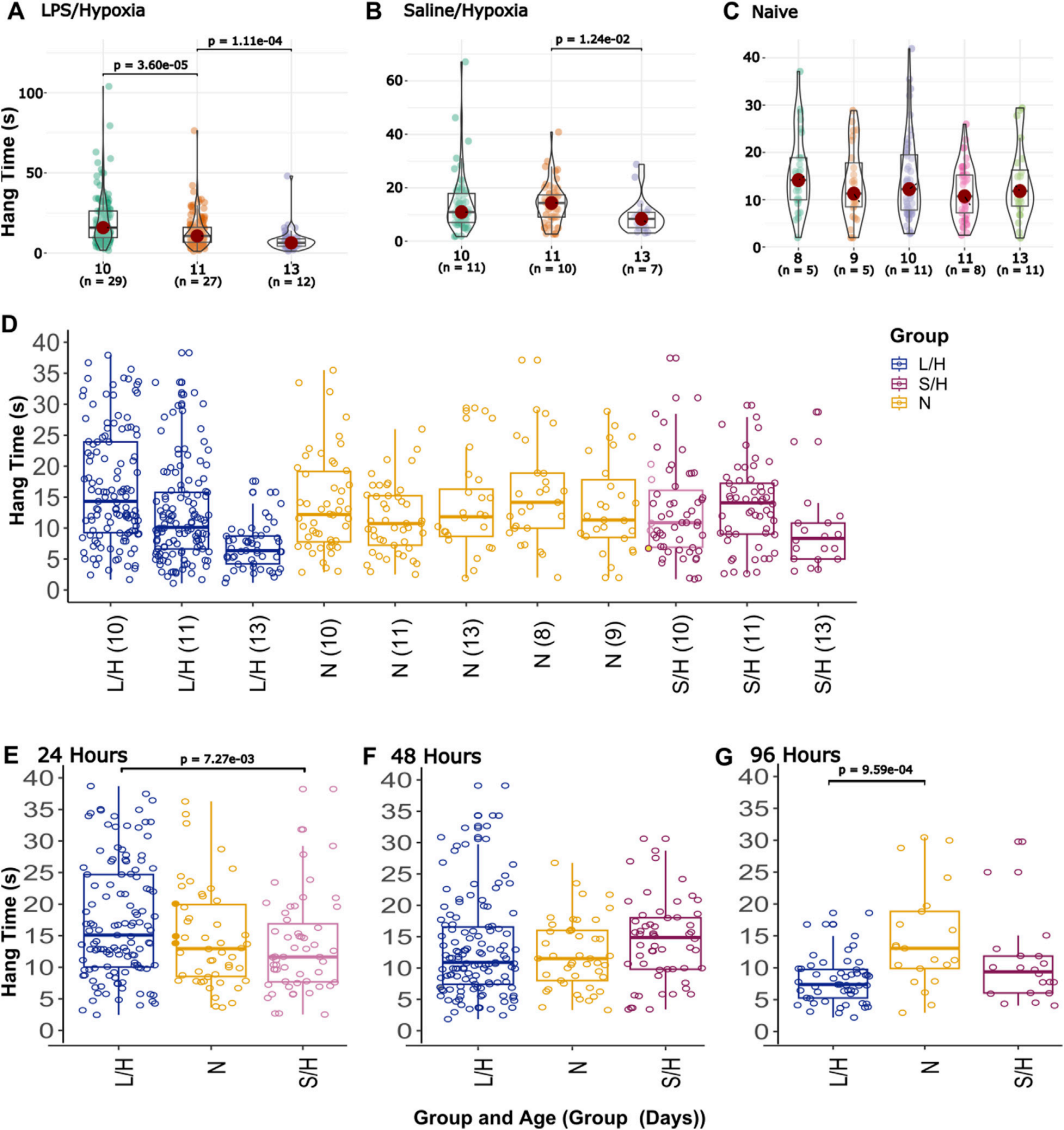
Hindlimb Hang data by group and gestational age. Group specific differences comparing changes within a group relative to age of the pups for **(A)** LPS/Hypoxia, **(B)** Saline/Hypoxia, and **(C)** Naïve. **(D)** The LH group showed significant differences at 48 and 96 (negative trend, A). The final section has each of the groups broken into different post-injury times 24 **(E)**, 48 **(F)**, and 96 **(G)**.

**FIGURE 7:**
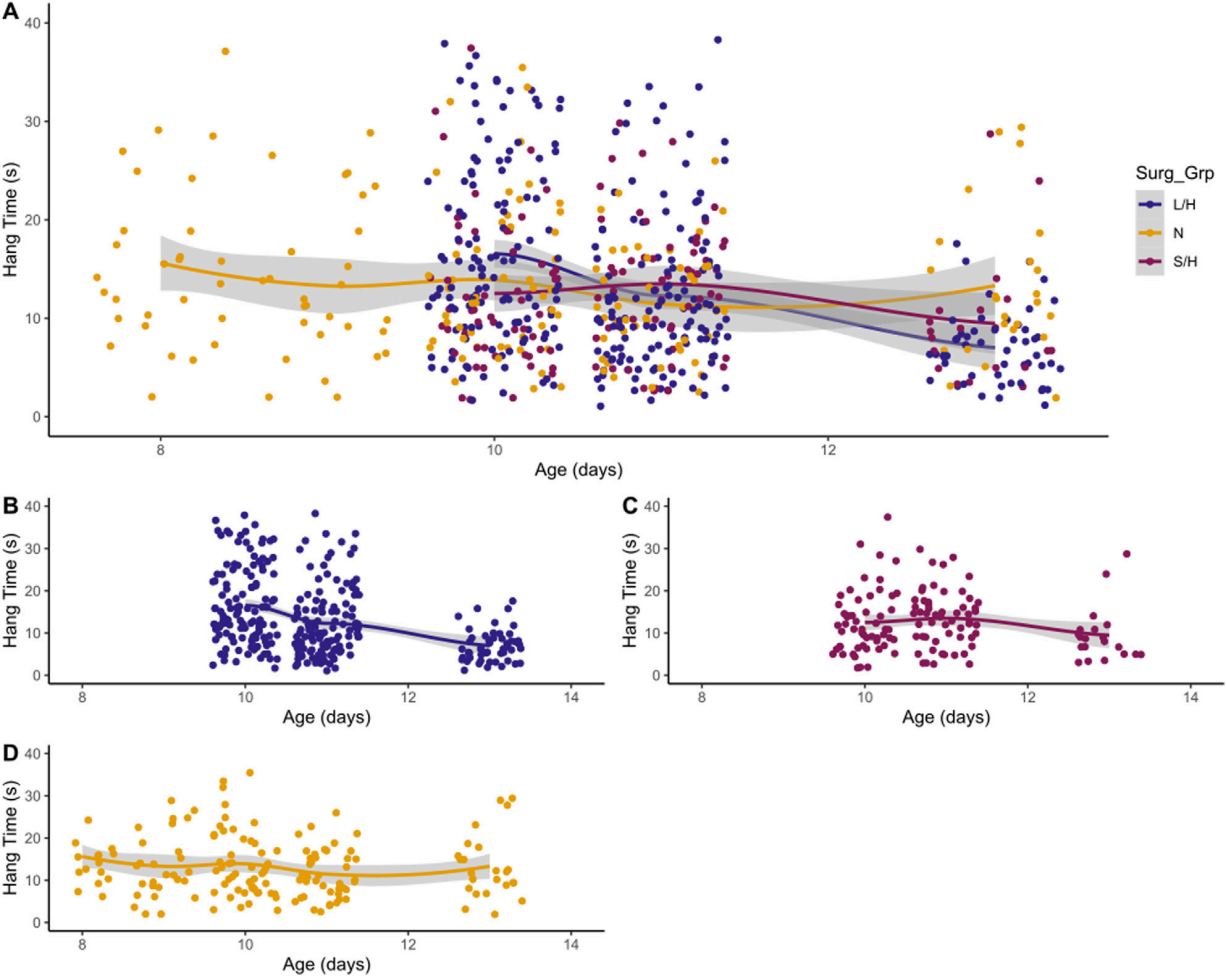
Hindlimb Hang vs. Age. While most groups show little change, **(C)** Saline/Hypoxia (S/H) and **(B)** LPS/Hypoxia (L/H) show decreased hang time. LPS/Hypoxia (L/H) animals show reduced hang time with increased age **(B)**. There is not a significant difference between overall hang times across all groups **(A)**. The **(D)** Naïve show more consistent trends with increasing age, neither decreasing or increasing in hang time.

**FIGURE 8:**
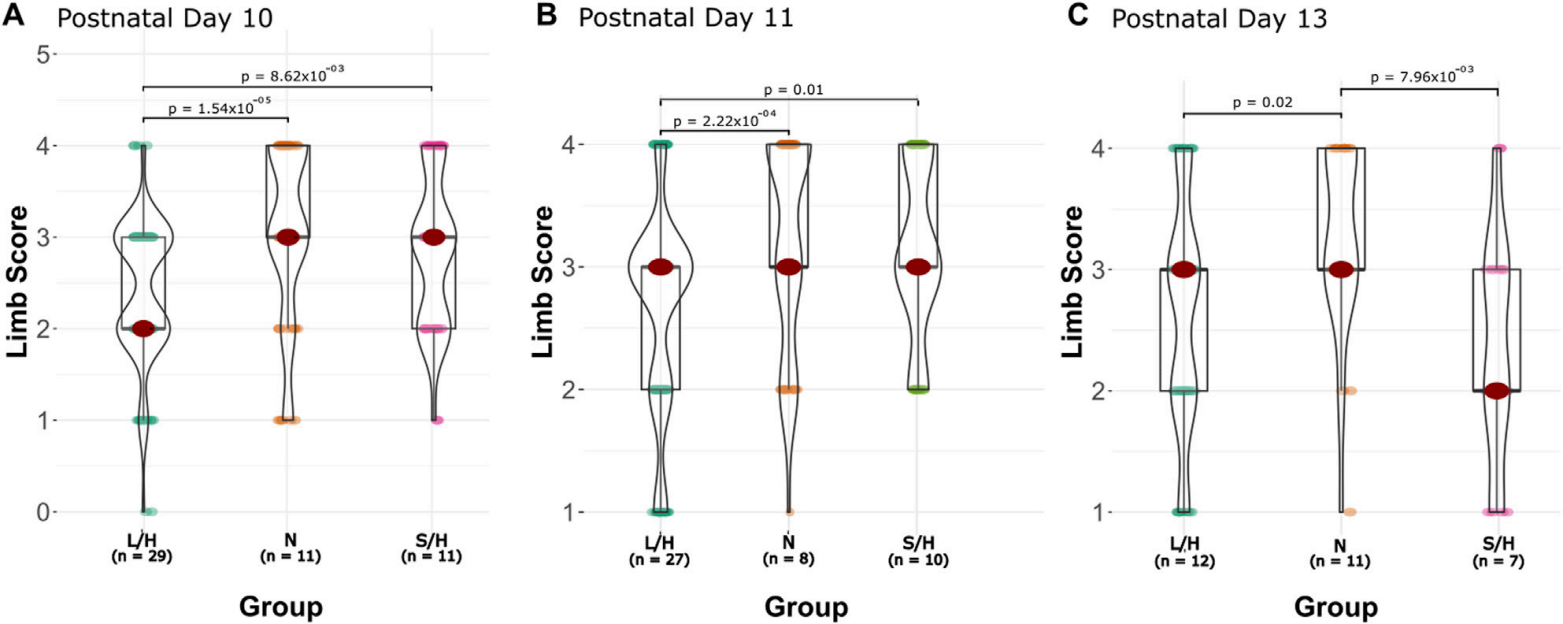
Hindlimb hang time by group and age. These protocols are based on protocols found in Feather and Ferguson, 2016. Limb score vs. age of the animals P10 **(A)**, P11 **(B)**, and P13 **(C)**. At age P10, LPS/Hypoxia animals have a significantly lower score than all other groups. At P11 all groups have the same median values; however LPS/Hypoxia (L/H) is significantly different from the other groups.

LPS/Hypoxia and Saline/Hypoxia animals showed a decreasing trend at 96 h. LPS/Hypoxia showed significantly shorter hang times at P10 to P11 (20.0 ± 15.6 vs. 13.0 ± 10.0, p-value = 3.60 × 10^−5^) and P11 compared to P13 (13.0 ± 10.0 vs. 7.8 ± 6.7, p-value = 1.11 × 10^−4^) hours (Figure 6A). Saline/Hypoxia showed similar trends but less extreme to LPS/Hypoxia, with the Saline/Hypoxia at P11 only showing a significant difference between the P11 and P13 (14.0 ± 7.4 vs. 9.5 ± 6.5, p-value = 1.24 × 10^−2^) hang time (Figure 6B). For the hindlimb hang test (Figures 6, 7), the Naïve group display consistent hang times (Figure 6). When we considered the difference between groups at P10, we found significant differences between LPS/Hypoxia and Saline/Hypoxia (20.0 ± 15.5 vs. 14.1 ± 11.2, p-value = 7.27 × 10^−3^). At P13 we saw significant differences between LPS/Hypoxia and Naïve (7.8 ± 6.7 vs. 13.3 ± 7.7, p-value = 9.59 × 10^−4^) were significant different at P13. The final comparison we considered was the difference between post-insult group-to-group comparisons. Additionally, when we considered limb score LPS/Hypoxia had a significantly lower score at P10 and P11 when compared to all other groups (Figure 8). This indicated that the LPS/Hypoxia animals were not as capable of holding their hind paws at a 90-degree angle to the lip of the container but instead had their legs come together in the center. In contrast, the other groups had higher scores indicating they were supporting their hind legs and holding on with strength to the side of the container.

### 3.3 IHC

#### 3.3.1 Caspase-9 staining

**FIGURE 9:**
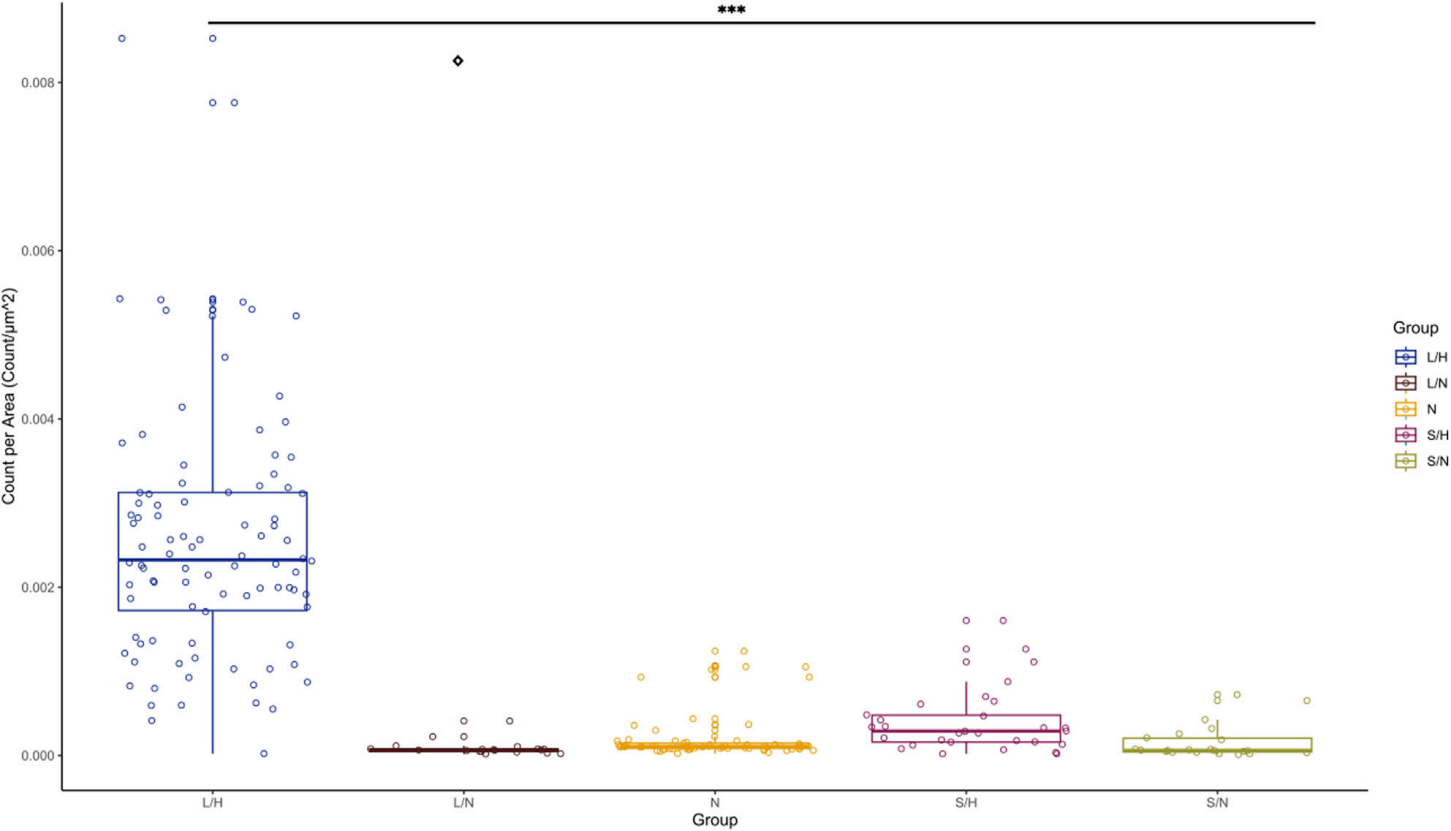
Caspase-9 expression in cortex. LPS/Hypoxia (n = 12), LPS/Normal Oxygen (n = 11), Naïve (n = 11), Saline/Hypoxia (n = 11) and Saline/Normal Oxygen (n = 9). Caspase-9 expression was significantly greater in the cortex of neonatal mouse brains in the maternal LPS/Hypoxia group when compared to all other groups (p-value <2.9e-9, Represented by ***). The saline/hypoxia group had statistically higher expression than LPS/normal oxygen (p-value of 1.7 × 10^−2^). As a result, saline/hypoxia would make the best SHAM animal when looking at the impacts of maternal inflammation and neonatal hypoxia. Significance is represented by a symbol with * indicating significance lower to LPS/Hypoxia. The p-values are one symbol = 0.05, two = 0.01, and three <0.001, ◊ indicate significance between Saline/hypoxia to LPS/normoxia.

Caspase-9 staining was used to label cells undergoing apoptosis due to the consequence of IL-1β and inflammation. Caspase-9 was used to help compare all possible control groups, Saline/Hypoxia was selected as the SHAM group for the remaining experiments. The following combinations of injections and hypoxia levels were conducted to determine the best option: LPS injection with normal oxygen (LPS/Normoxia) from P3 to P10, saline injection with hypoxia (Saline/Hypoxia) (10% oxygen) from P3 to P10, and saline injection with normal oxygen (Saline/Normoxia) from P3 to P10. Sections were quantified utilizing the Stereologer program and normalized to the cortex area. When comparing the groups using nonparametric statistical analysis (Kruskal–Wallis with Dunn *post hoc* test), the LPS/Hypoxia group showed significantly higher caspase-9 expression per cortex area than any other group (p-value <2.42 × 10^−8^). Additionally, the LPS/Normoxia group demonstrated significantly lower expression than Saline/Hypoxia (p-value = 2.27 × 10^−2^) (Figure 9). There were no significant differences between the other groups. Due to elevated caspase-9 expression in the Saline/Hypoxia animals, the Saline/Hypoxia animals were SHAM controls for succeeding experiments.

**FIGURE 10:**
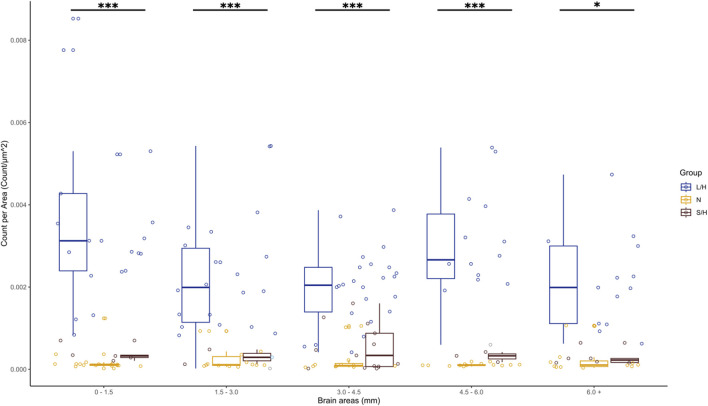
Caspase-9 expression was significantly higher in LPS/Hypoxia group (L/H) compared to all other groups in each brain section. There was no significant difference in caspase-9 expression between the Naive or Saline/Hypoxia (S/H) groups. The p-values are one symbol = 0.05, two = 0.01, and three <0.001.

Additionally, we determined if there were differences in caspase-9 stain throughout the LPS/Hypoxia brains. By examining the staining in a block of 1.5 mm of the brain, we found that caspase-9 expression in the cortex of LPS/Hypoxia animals was not significantly different in any one area of the brain in either LPS/Hypoxia animals or Naïve control animals. When analyzing the longitudinal expression of caspase-9 between Saline/Hypoxia, LPS/Hypoxia, and Naïve animals, we found that the LPS/Hypoxia animals have statistically higher expression of caspase-9 for all five brain regions when compared to the other groups (p-value <0.001). However, there is no significant difference between any brain regions’ expression of caspase-9 within each group (Figure 10).

#### 3.3.2 MAP2

**FIGURE 11:**
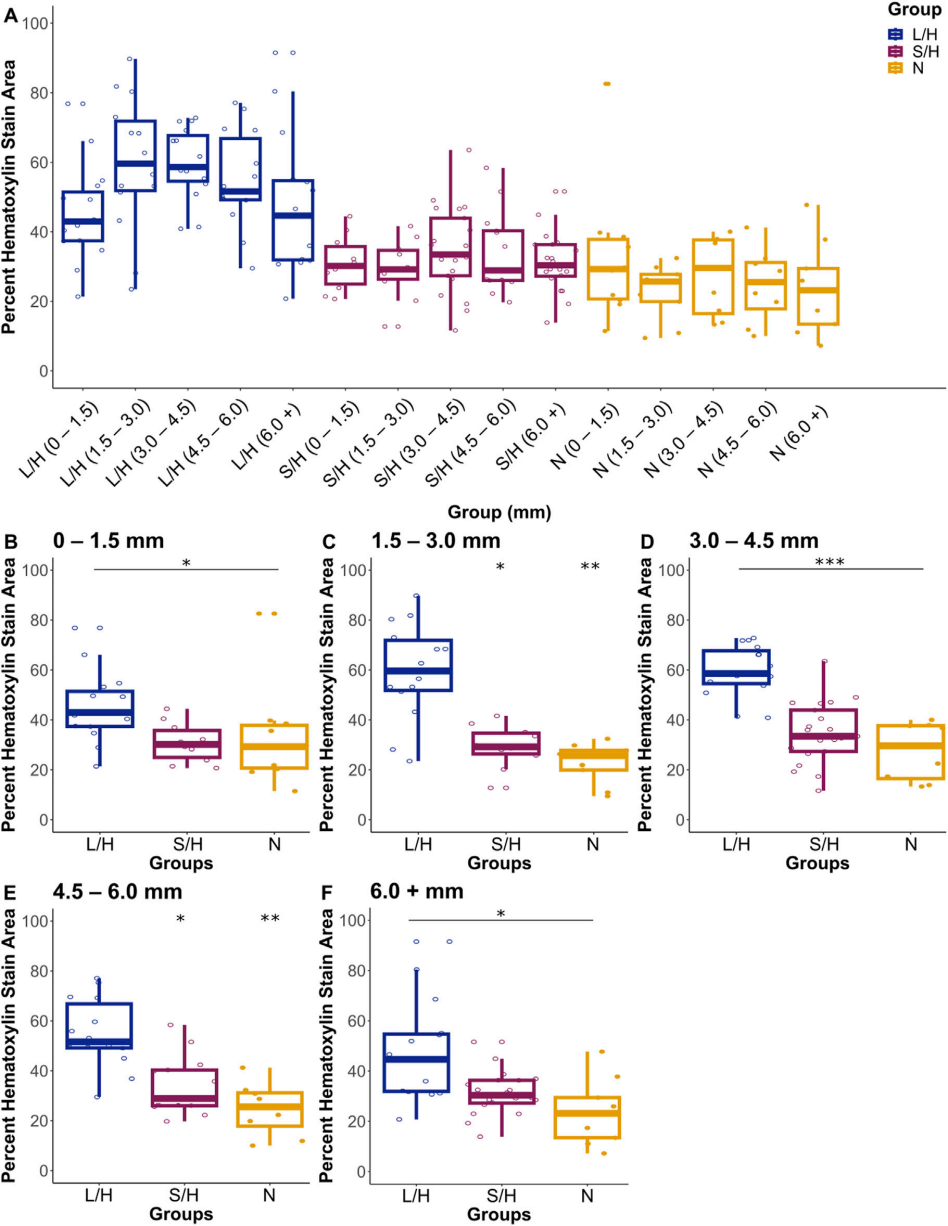
Hematoxylin stain area by group. Three treatment groups show differences in area of damage from rostral to caudal **(A)**. The brains were then subdivided into smaller graphs to compare levels in each area; A1 = 0–1.5 mm **(B)**, A2 = 1.5–3.0 mm **(C)**, A3 = 3.0–4.5 mm **(D)**, A4 = 4.5–6.0 mm **(E)** and A5 = 6.0 + mm **(F)**. Significance is relative to LPS/Hypoxia, p-values: * 0.05, ** 0.01 and *** 0.001.

**FIGURE 12:**
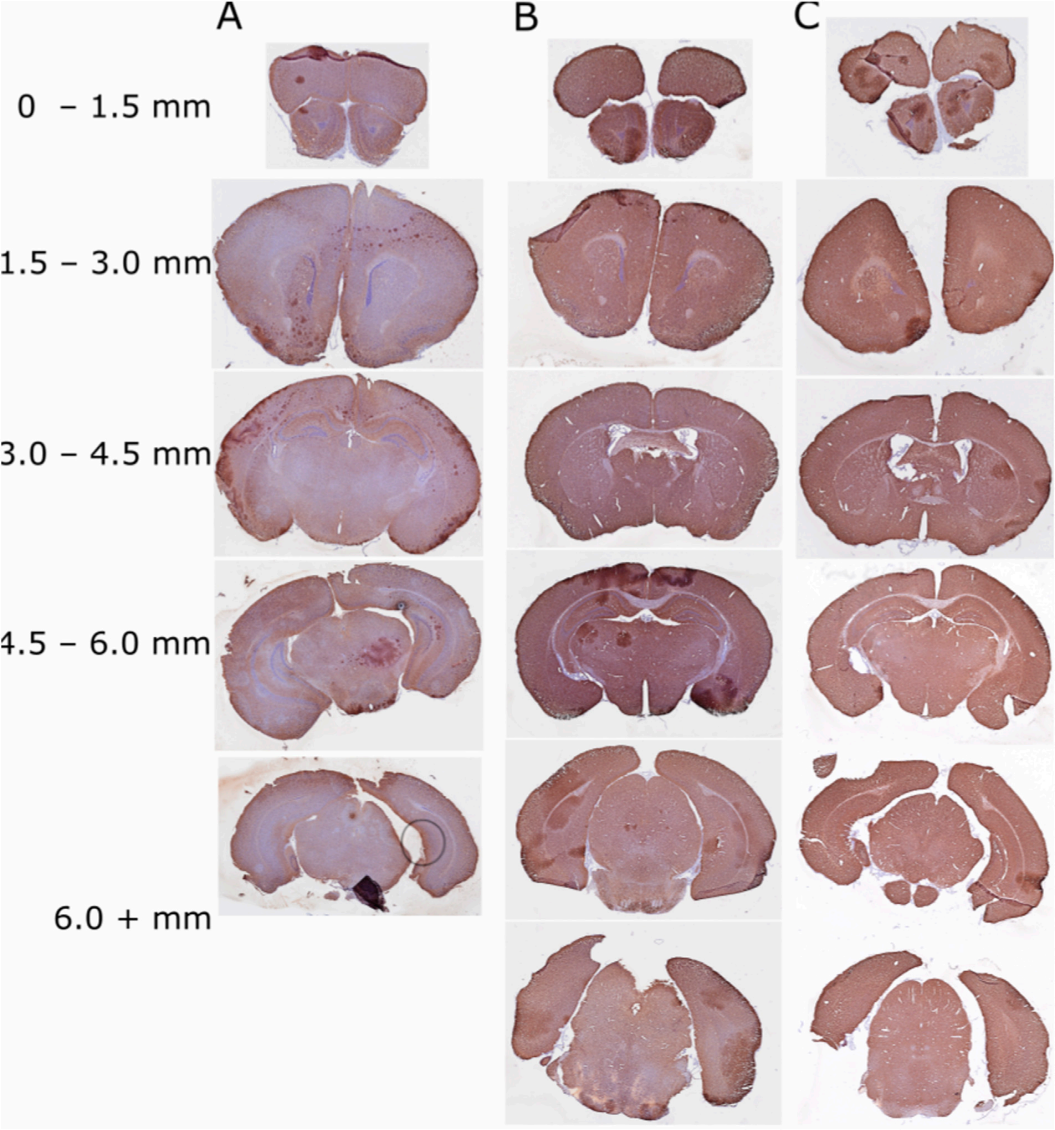
Photomicrographs of each group:LPS/Hypoxia **(A)**, Saline/Hypoxia **(B)** and Naïve **(C)** stained with MAP2 (Brown) and counterstained with hematoxylin (Purple/blue). All slides show the difference in areas represented in Figure 11.

In addition to caspase-9, we quantified changes in MAP2. Previous studies demonstrated that MAP2 expression is similar to that of TTC staining in showing areas of damage (Kharlamov et al., 2009; Popp et al., 2009). We stained slides and quantified the area of MAP2 stain and area of Hematoxylin stain within five areas of the brain (A1 = 0–1.5 mm, A2 = 1.5–3.0 mm, A3 = 3.0–4.5 mm, A4 = 4.5–6.0 mm and A5 = 6.0 + mm, rostral to caudal starting with the end of the olfactory bulb and first appearance of the cortex) (Figure 11). For the most rostral area of the brain (A1, 0–1.5 mm), the LPS/Hypoxia brain had a significantly higher percentage of hematoxylin area when compared to that of the Naïve (p-value = 2.63 × 10^−2^) and Saline/Hypoxia (p-value = 4.24 × 10^−2^). In A2, (1.5–3.0 mm) of the brain, we found that the LPS/Hypoxia brains had significantly higher percentages of hematoxylin than all other groups (N of 8 for each group, p-value = 1.46 × 10^−4^, and Saline/Hypoxia p-value = 1.43 × 10^−2^). In A3, (3.0–4.5 mm) and A4, (4.5–6.0 mm), LPS/Hypoxia animals had higher hematoxylin area when compared to Naïve (p-value = 9.82 × 10^−5^, p-value = 6.67 × 10^−4^) and Saline/Hypoxia (p-value = 4.35 × 10^−5^, p-value = 5.30 × 10^−3^) animals. In the most caudal area (A5, 6.0 + mm), LPS/Hypoxia animals had a significantly higher area of hematoxylin than Naïve (p-value = 2.30 × 10^−3^) animals but no significant difference between Saline/Hypoxia (p-value = 3.14 × 10^−2^) (Figure 12).

### 3.4 qPCR

**FIGURE 13:**
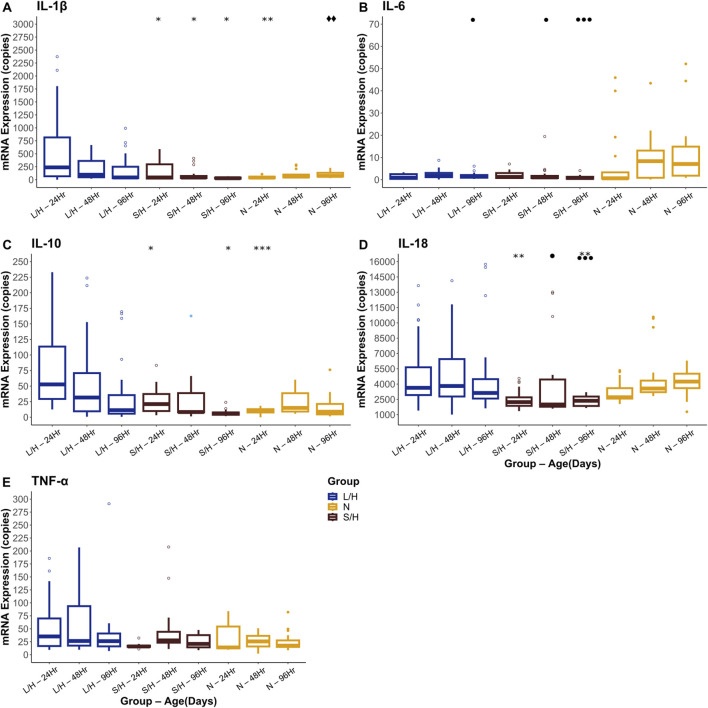
Quantification of qPCR for IL-1β **(A)**, IL-6 **(B)**, IL-10 **(C)**, IL-18 **(D)**, and TNF-α **(E)**. Significance is represented by the significantly higher group LPS/Hypoxia (*), Saline/Hypoxia (⧫), and Naïve (●). Significance levels are represented by the number of symbols with p-values: * 0.05, ** 0.01 and *** 0.001.

**FIGURE 14:**
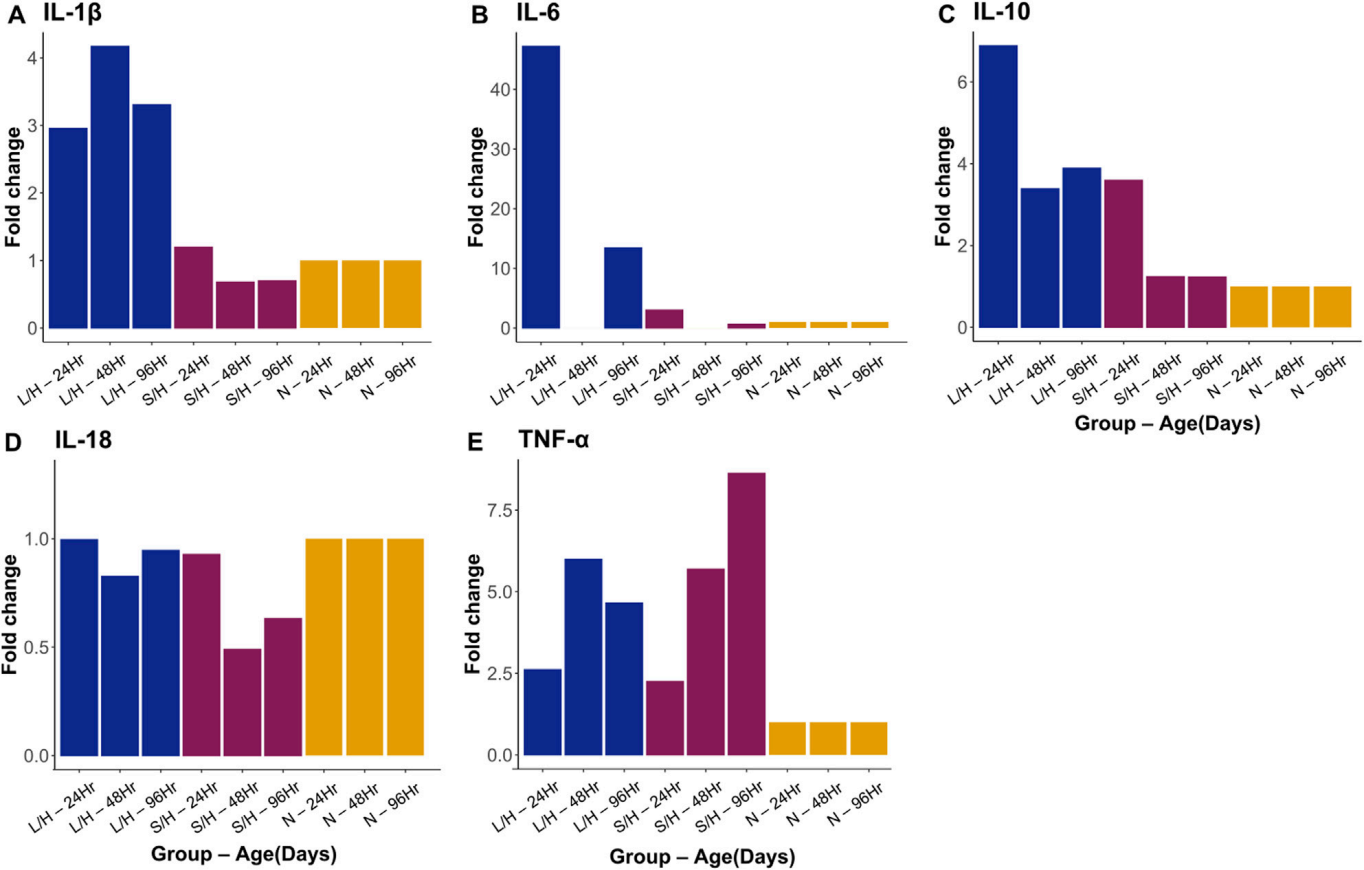
Fold change for qPCR data looking at IL-1β **(A)**, IL-6 **(B)**, IL-10 **(C)**, IL-18 **(D)** and TNF-α **(E)**. While different trends were observed there was no statistically significance when compared with Krustal-Wallis and Dunn tests.

IL-1β expression showed significant changes in absolute quantification across several groups at different time points. When examining all five groups at 24 h post-injury, we noticed that LPS/Hypoxia animals had significantly higher expression than Naïve (p-value = 7.27 × 10^−4^), and Saline/Hypoxia animals (p-value = 0.01). LPS/Hypoxia was significantly higher than Saline/Hypoxia group at 48 (p-value = 2.01 × 10^−2^) and 96 h (p-value = 1.07 × 10^−2^) groups. In contrast, it is important to note that the median of Naïve animals at 96 h was higher than all other groups. However, this is not significant (Figures 13A, 14A).

IL-6 expression was significantly different at different expression levels. At 96 h post-injury, LPS/Hypoxia animals showed a significant decrease in expression when compared to Naïve (p-value = 5.73 × 10^−3^) animals. Naïve animals had higher expression than Saline/Hypoxia (p-value = 2.70 × 10^−2^) animals at 96 h as well (Figures 13B, 14B).

At 24 h post-injury, we saw significantly higher expression IL-10 in LPS/Hypoxia animals when compared to Naïve (p-value = 5.27 × 10^−7^), and Saline/Hypoxia (p-value = 5.03 × 10^−3^) animals. At 96 h we saw significantly higher expression of LPS/Hypoxia animals when compared to Saline/Hypoxia (p-value = 4.22 × 10^−2^) animals (Figures 13C, 14C).

The next gene of interest was IL-18. At 24 h post-injury, there was a significantly higher expression of LPS/Hypoxia (p-value = 4.34 × 10^−4^) when compared to Saline/Hypoxia and Navie (p-value = 5.27 × 10^−7^). At 96 h post injury LPS/Hypoxia had significant different expression from Saline/Hypoxia (p values 4.22 × 10^-^) were significantly higher than Saline/Hypoxia animals (Figures 13D, 14D).

Our final target of interest was TNF-α expression at 24, 48, and 96 h post-injury. There was no significant difference between any of the groups at any time point for TNF-α expression (Figures 13, 14).

### 3.5 Protein expression

**FIGURE 15:**
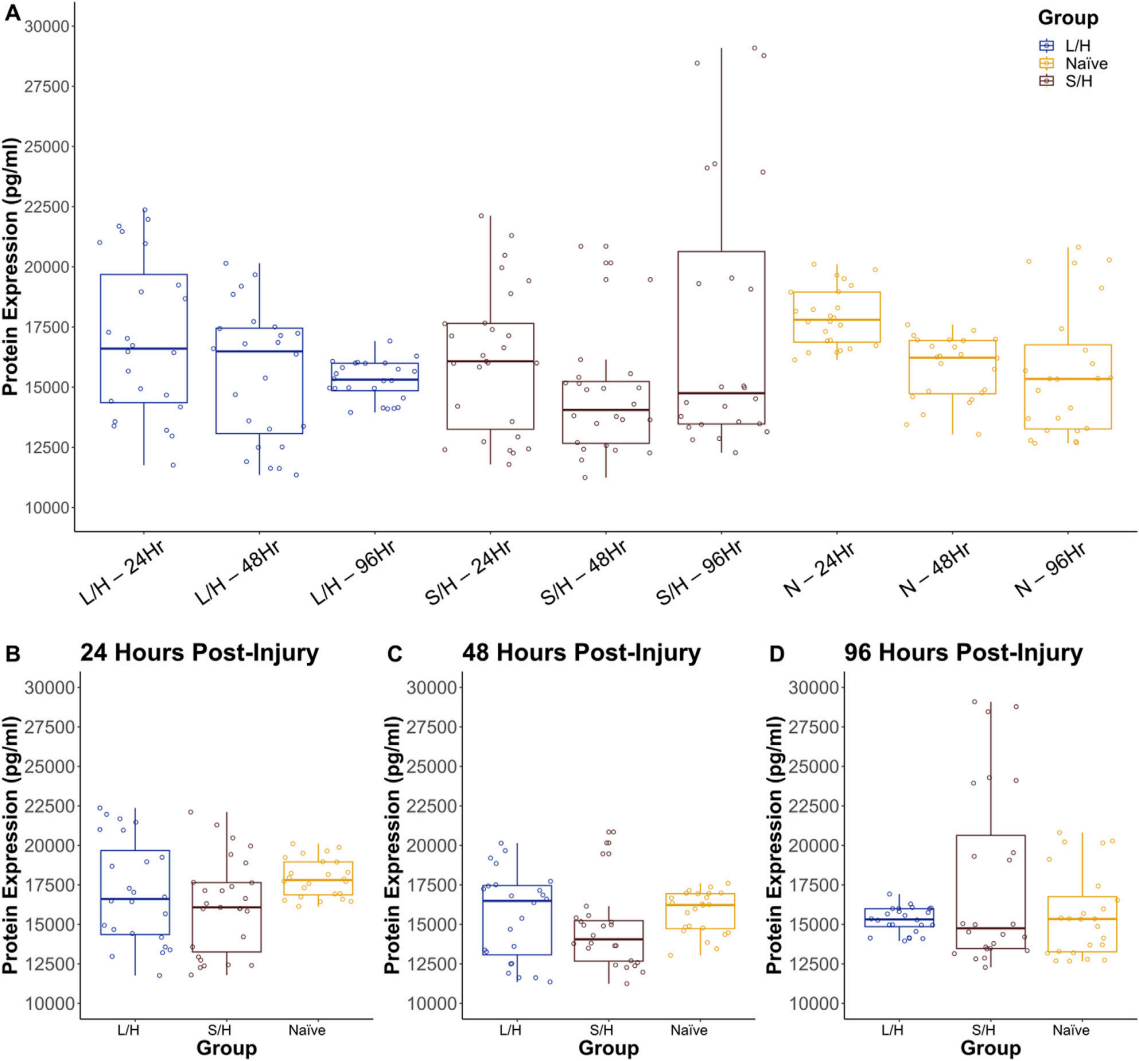
ELISA of IL-18 expression in LPS/Hypoxia, Naïve, and Saline/Hypoxia animals **(A)** at 24 **(B)**, 48 **(C)**, and 96 **(D)** hours post-injury. There was no statistically significant difference between the groups.

To validate the PCR reactions protein quantification was conducted utilizing ELISAs. The ELISA showed that there was no significant difference in IL-18 expression at any time point or in any group (Figure 15).

## 4 Discussion

In this study, we tested the hypothesis that a mouse model of hypoxic-ischemic encephalopathy (HIE) incorporating maternal factors (including stress and inflammation) more closely replicates human HIE than prior animal models. HIE refers to a specific set of conditions where perinatal hypoxic ischemia is the primary cause of injury in the newborn, however, this is difficult to confirm in practice (Molloy and Bearer, 2018). The model we used encompasses the timeline for preterm HIE with early onset inflammation (Figure 2). In the context of HIE, a major relatively poorly researched factor is maternal state and any role that maternal stress or inflammatory tone plays in predisposing the fetus to hypoxic-ischemic injury.

Commonly used murine models for HIE do not incorporate maternal factors, their impact on preterm HIE (pHIE) and, just as importantly, do not replicate the patterns of ischemic damage seen in human infants. Others have pointed out this gap in rodent models of HIE (Bennet et al., 2003; Guo et al., 2010). As a result, the limitations of current HIE therapies may be exacerbated by this mismatch between murine models and human disease. Longitudinal studies of learning and development in preterm infants receiving therapy targeting HIE, like hypothermia, do not show improvement in outcome compared to those who had not received the therapy. Also, many therapies are not FDA-approved for early preterm neonates (Cainelli et al., 2021). The model we used, thus, was designed to incorporate maternal factors and murine models incorporating fetal-maternal interactions likely provide a better foundation for development of therapy for patients with HIE.

Infants suffering from preterm hypoxic-ischemic encephalopathy can present with a wide range of disabilities and physical indicators of HIE. Diagnosis of preterm hypoxic-ischemic encephalopathy (pHIE) is not simple and varies depending on location (Gopagondanahalli et al., 2016) and severity of injury. Premature infants are scored using several basic health assessment scales. The APGAR scale which targets scores below 5 at 1 min following birth and a score below 7 at 5 min following birth (Schmidt and Walsh, 2010; Logitharajah et al., 2009) is commonly used for pulse, irritability, muscle tone, and respiration. Additional markers used in the clinic are blood pH and base deficits in the blood (Schmidt and Walsh, 2010). A pH less than 7 as well as a deficit in the blood of 15 mmol/L is a strong indicator that further tests need to be performed to determine if HIE has occurred (Schmidt and Walsh, 2010; Herrera et al., 2018). The care team scores the severity of HIE based on the Sarnat staging (Gopagondanahalli et al., 2016) which is specific to HIE. Reports vary regarding the long-term consequences of pHIE, from no neurological deficits to severe outcomes such as cerebral palsy (Chalak et al., 2014; Gopagondanahalli et al., 2016; Logitharajah et al., 2009). Because of the variability of HIE presentation, it is important to have a model that physiologically resembles human pHIE/HIE so we can better understand the underlying mechanisms and pathways affected by hypoxic-ischemic injury and develop targeted interventions to reduce the severity of HIE.

An important factor to consider when evaluating any model is its relevance to human patients. In human pHIE, markers that are correlated with pHIE are low body mass index and abnormal APGAR scores (Douglas-Escobar and Weiss, 2015; Schmidt and Walsh, 2010). While there is no comparable early life assessment such as an APGAR score in mice, we can look at the physiology and behavior of animals to evaluate their physical condition. Two key physiological characteristics that can be non-invasively observed and scored are weight and length—the LPS/Hypoxia treatment groups were well below that of naïve animals. Linear regression showed a significant difference between treatment groups’ age, weight, and length (Figure 3). In this study, we found that the LPS/Hypoxia animals showed similar morphologic trends to those seen in humans, with reduced birth weight and reduced growth rate (Figure 4). Behavioral data was not significantly different between experimental groups however the LPS/Hypoxia animals performed poorly in tests that required strength or muscle mass (Figures 5, 6), validating our observations from the other physiology data.

Inflammation can directly lead to the activation of caspase dependent apoptosis and further damage in the brain of preterm infants. Once the pro-inflammatory pathway is activated, IL-1β binds with IL-1R, and TNF-α activates TNFR, leading to an increase in expression of Caspase-9 (He et al., 2022). Once activated, caspase-9 cleaves caspase-3 which results in the creation of apoptosomes in the cell membrane (He et al., 2022). Continued activation of cytokines and increased cell death activates microglia, and the release of pro-inflammatory cytokines generates a chronic state of inflammation in the brain of preterm infants (Kaushik et al., 2013). Common therapies designed to target inflammation and damage in the brain are considered to be ineffective in preterm infants (Bennet et al., 2003). As a result, individuals with pHIE can experience prolonged neural inflammation throughout their lives (Guo et al., 2010).

To assess the role of maternal and fetal inflammation in this model in pHIE, we looked at two important damage markers using immunohistochemistry. We chose caspase-9 because it is downstream from IL1R activation becauase it provided an ideal marker for indicating cells moving towards apoptosis (He et al., 2022). Another commonly used marker of ischemic injury is TTC (2,3,5-Triphenyltetrazolium chloride). TTC staining has been used in RV models of HIE, however, we chose MAP2 as it has been shown to decrease expression following ischemic injury seen in middle cerebral artery occlusion (MCAO) and RV models (Kharlamov et al., 2009; Popp et al., 2009). Our treatment groups, LPS/Hypoxia, LPS/Normoxia, Saline/Hypoxia, Saline/Normoxia and Naïve groups were stained for Caspase-9 to look at the damage from the different exposure. When we quantified expression of Caspase-9 in the brain of LPS/Hypoxia, LPS/Normoxia, Saline/Hypoxia, Saline/Normoxia, and Naïve mice pups we observed that LPS/Hypoxia animals had significantly greater staining in the cortex compared to the other treatment groups. These results showed that the maternal derived model resulted in more severe outcomes in the pups and that the cytokine changes were not the result of hypoxia or maternal inflammation in isolation (Figure 14). We originally expected the LPS/Hypoxia animals would have lower caspase-9 staining in A4 – A6 zones but found more severe injury throughout the LPS/Hypoxia group.LPS/Hypoxia protocol results in diffuse and widespread damage—like the clinical presentation of HIE (Figure 12).

In addition to the structural markers of damage, we quantified expression of key pro-/anti-inflammatory cytokines including IL-1β, IL-6, IL-10, IL-18, and TNF-α. We quantified message expression using qPCR (Figure 14). We saw increased fold changes in IL-1β at all three time points (Figure 14A), and higher IL-10 message, particularly at 24 h (Figure 14B) with slight elevation at 48 and 96 h. Two unexpected outcomes were the increase in TNF-α (Figure 14E) and IL-18 (Figure 14D). We expected IL-18 to be greater, particularly at 96 h in LPS/Hypoxia animals (Figures 15C-D). However, all animals showed greater expression of IL-18. This was validated with ELISA (Figure 15A) with higher levels of IL-18 protein in all groups with no significant differences across treatments. TNF-α in the Saline/Hypoxia group increased and this is consistent with prior work showing that hypoxia increases TNF-α levels (Szaflarski et al., 1995; Sourial-Bassillious et al., 2006; Mukandala et al., 2016). When considering the role that hypoxia plays in TNF-α expression it is interesting that LPS/Hypoxia animals do not show an increase in expression over time. TNF-α and IL-1β showed prolonged, higher expression levels (Figures 14, 15) than the controls, suggesting that the LPS/Hypoxia animals are experiencing a longer inflammatory state similar to that seen in human preterm hypoxic-ischemic encephalopathy. This is further supported by the increase of 24-h post-injury IL-10 expression. Previous work on IL-10 in traumatic brain injury and HIE in mouse models shows that it plays an important anti-inflammatory role by reducing brain damage and decreasing pro-inflammatory signaling (Szaflarski et al., 1995; Sourial-Bassillious et al., 2006). When assessing IL-1β and IL-10 together we see opposite trends over time, as a result we may have missed the extreme upregulation of IL-1β and other pro-inflammatory markers which occur within a few hours after injury.

Our study has several limitations due to the nature of the model and available methodology. One limitation is variability in the molecular experiments, likely due to dilution of expression since we were using whole brains instead of more localized selection via tissue punches—effectively we are quantifying expression relative to the brain rather than local neuroinflammation and cell death. We used whole brains since the exact location of damage for the LPS/Hypoxia animals has not previously been described. Additionally, much like damage patterns seen in human infants, there are differences in location and extent of damage in animal models (Gunn and Bennet, 2009) so using the whole brain allowed us to characterize the damage in the model using broad strokes while setting the stage for more localized assessment in the future. Based on the caspase-9 and MAP2 expression, isolation of just cortex, following perfusion, would provide more specific targeting of expression of damage markers and cytokine expression (Figure 12). Due to the small size of mouse pups and lack of an appropriate method to assess blood flow, we have no direct measurements of changes in CNS perfusion, thus no direct way to confirm localized ischemia. Obvious future directions include use of small animal imaging (MRI and ultrasound) and high-resolution detection of blood-borne biomarkers (e.g., multiplex and RNAscope) to further characterize this model in comparison to human HIE.

The shorter gestation period and earlier developmental stage of neonates in murine models provide a good approximation of prematurity in human infants. Yet, there are still differences between preterm humans and neonatal mice that make it difficult to generate a model incorporating maternal factors influencing preterm hypoxic-ischemic encephalopathy. Because we had a lengthy gap between initiation of inflammation and hypoxia challenge, we observed blunted expression of early onset proinflammatory cytokines. We plan to conduct additional studies to look at the levels of cytokines at different stages of the experimental protocol including removing some animals earlier than P9 to see if we can detect higher levels of cytokine expression.

In the future, we are considering treating dams with anti-inflammatory drugs to reduce the maternal inflammation and see if it will improve the pups’ outcomes. Additional follow-up experiments will include a more detailed survey of changes in pro- and anti-inflammatory markers in the dams. Finally, investigation into why neonates in this model show prolonged inflammation and are unable to arrest the inflammatory upregulation would be useful in understanding pHIE.

## 5 Conclusion

Based on our results, the LPS/Hypoxia mouse model of pHIE provides a maternally derived inflammation model that shows the critical importance of maternal-fetal communication in perinatal ischemic injury. The model we used has more diffuse HIE injury, like that seen in the clinical presentation of HIE in human infants, and incorporates maternal inflammation and stressors in ways that is absent in other models of HIE and ischemic encephalopathy in neonates. By assessing the effects of multiple inflammatory cytokines, changes in expression and cell death the connection between maternal induced injury and fetal neural damage can provide nascent information to better understand fetal-maternal interactions in this model. We suggest that use of this model will help other investigators focused on multi-scalar aspects of fetal and neonatal ischemic injury, from cellular responses, to neurodevelopmental differences, and whole organism behavior in response to maternal stress and inflammation. The prolonged inflammatory state, extent and pattern of damaged tissue in the brain, and activation of executioner caspases, all indicate that this model exhibits damage that is beyond that seen with a short-term hypoxic insult and shows similarities to clinical outcomes observed in neonates diagnosed with pHIE. We have taken a systems biology approach by quantifying cellular level damage, changes in message, overall quantification of CNS damage, assessing neurodevelopmental impairment via behavioral tests, and quantified gross changes in morphology in the whole animal. A novel aspect of this work is inclusion of maternal stress and its impact on the neonatal organism. This model provides a foundation for further studies on maternal-fetal interactions in hypoxic-ischemic injury and can be used to develop new therapeutic approaches to mitigate perinatal HIE and improve outcomes.

## Data Availability

The datasets presented in this study can be found in online repositories. The names of the repository/repositories and accession number(s) can be found below: https://github.com/drcgw/Front-SystemsBiology-1517712.
