# Supplementary figures and images for: Inflammation mediated brain damage and cytokine expression in a maternally derived murine model for preterm hypoxic-ischemic encephalopathy
Source: Front Syst Biol. 2025 Jul 1;5:1517712. doi: 10.3389/fsysb.2025.1517712 (PMC12342018; doi:10.3389/fsysb.2025.1517712)

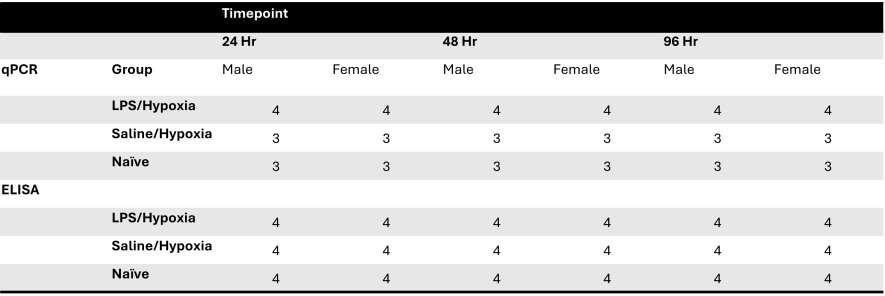

Supplement: Supplementary file 1 [file Image3.tiff]

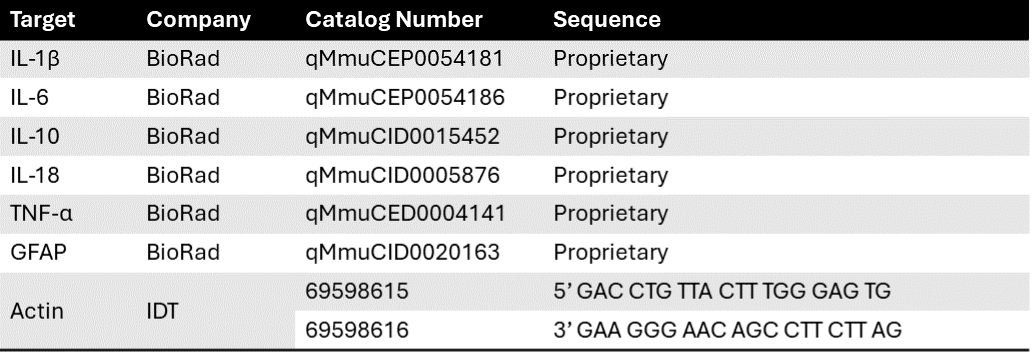

Supplement: Supplementary file 2 [file Image1.tiff]

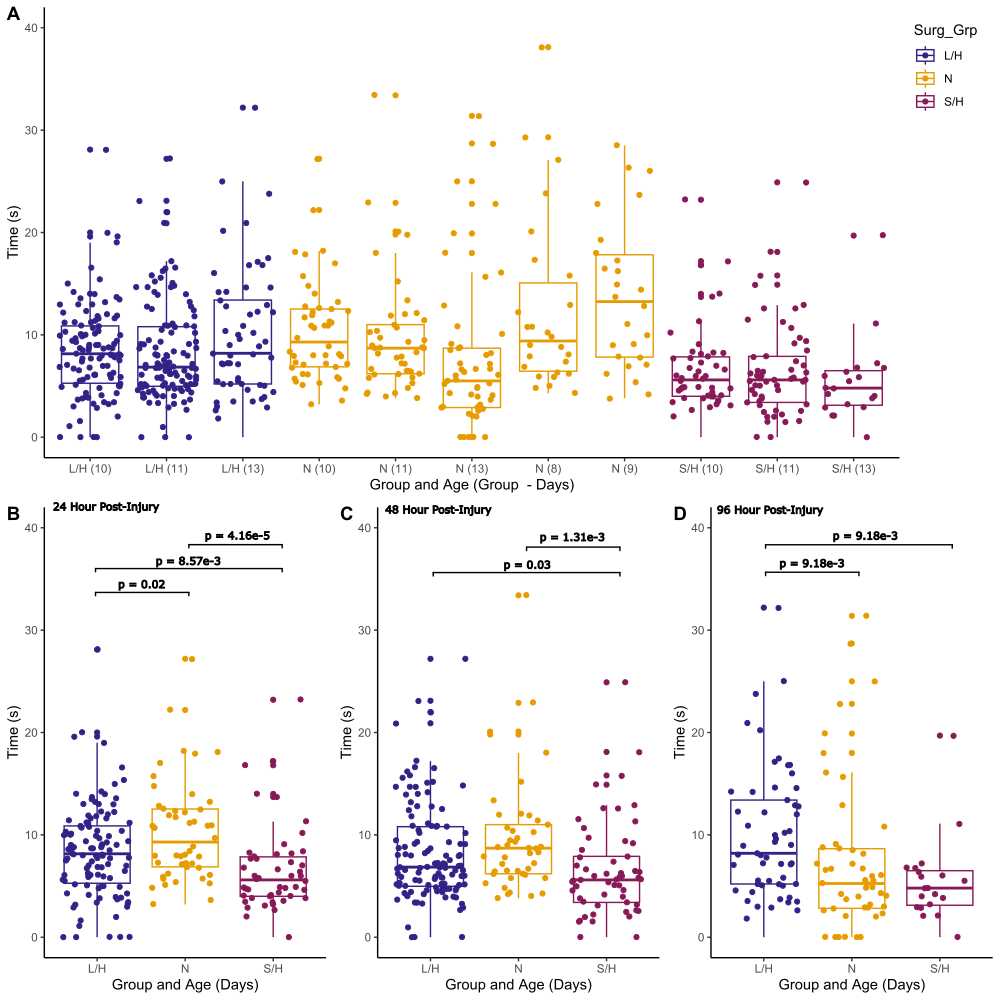

Supplement: Supplementary file 3 [file Image9.tiff]

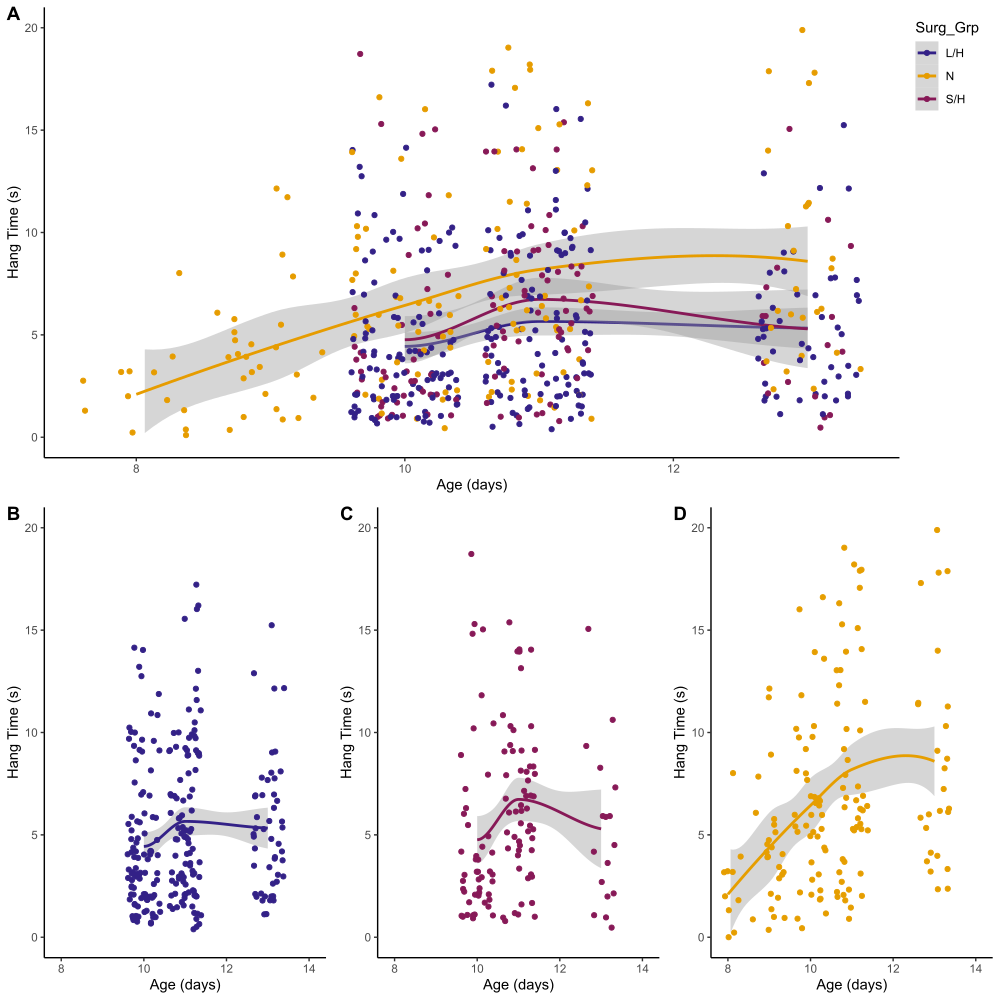

Supplement: Supplementary file 4 [file Image13.tiff]

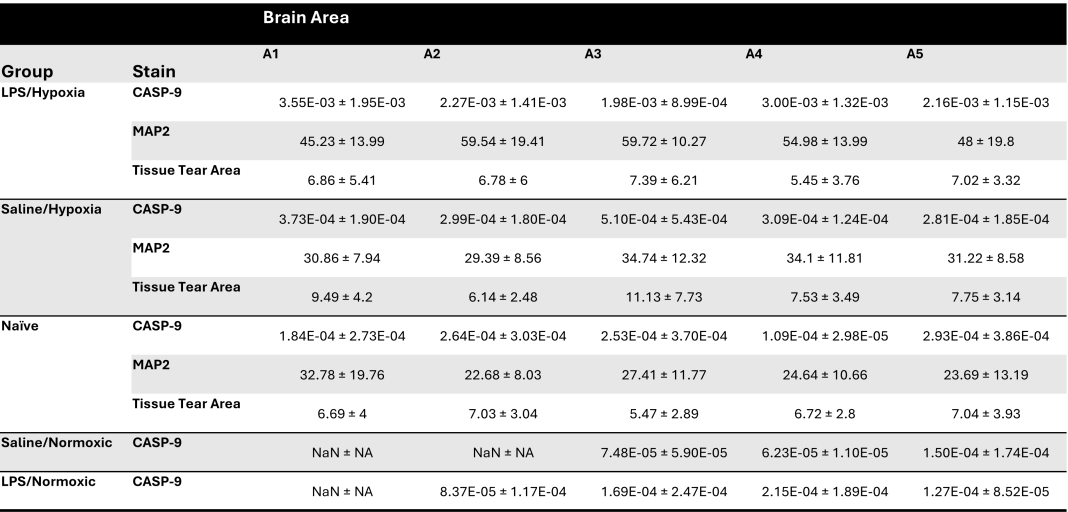

Supplement: Supplementary file 5 [file Image5.tiff]

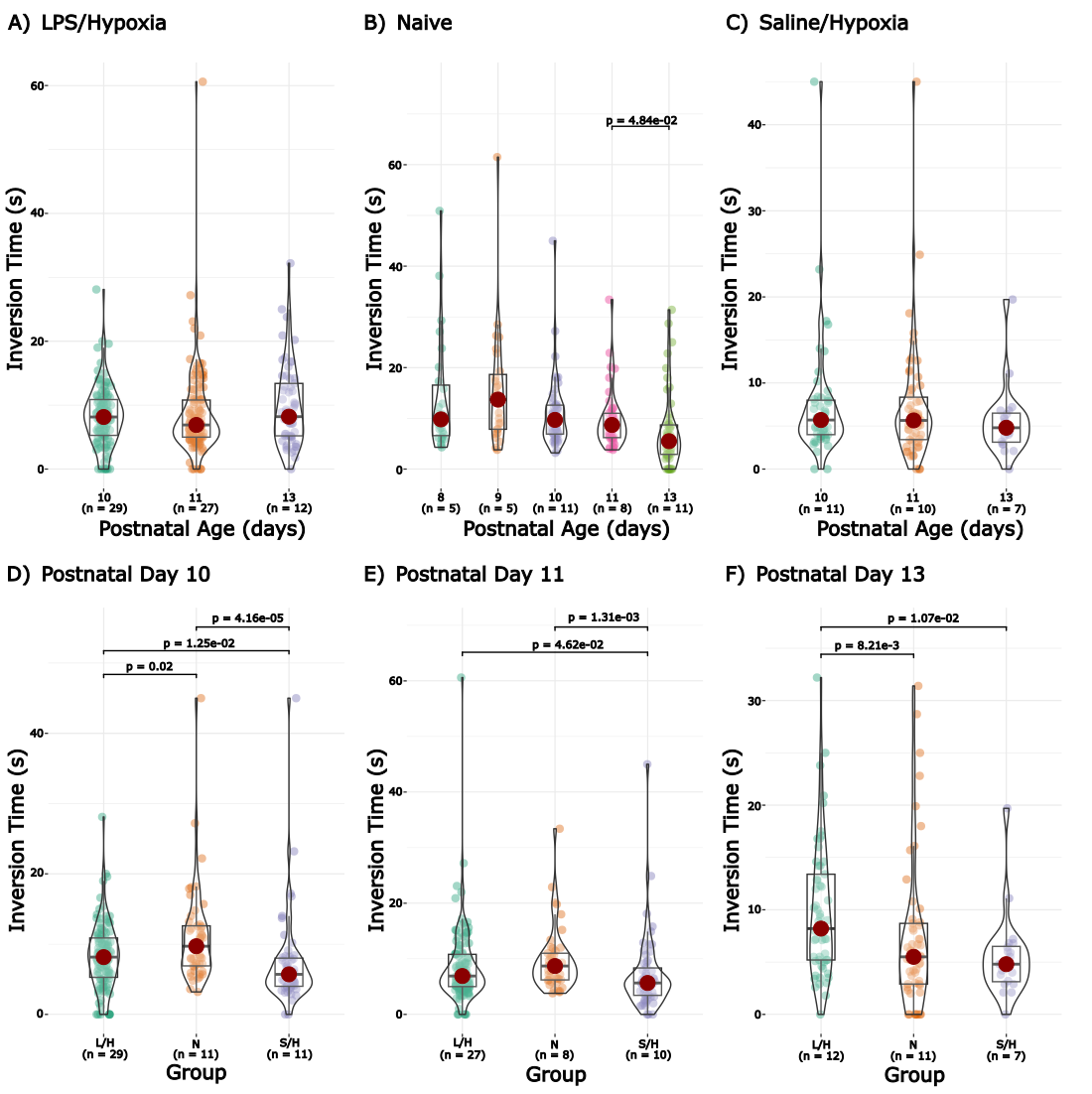

Supplement: Supplementary file 6 [file Image8.tiff]

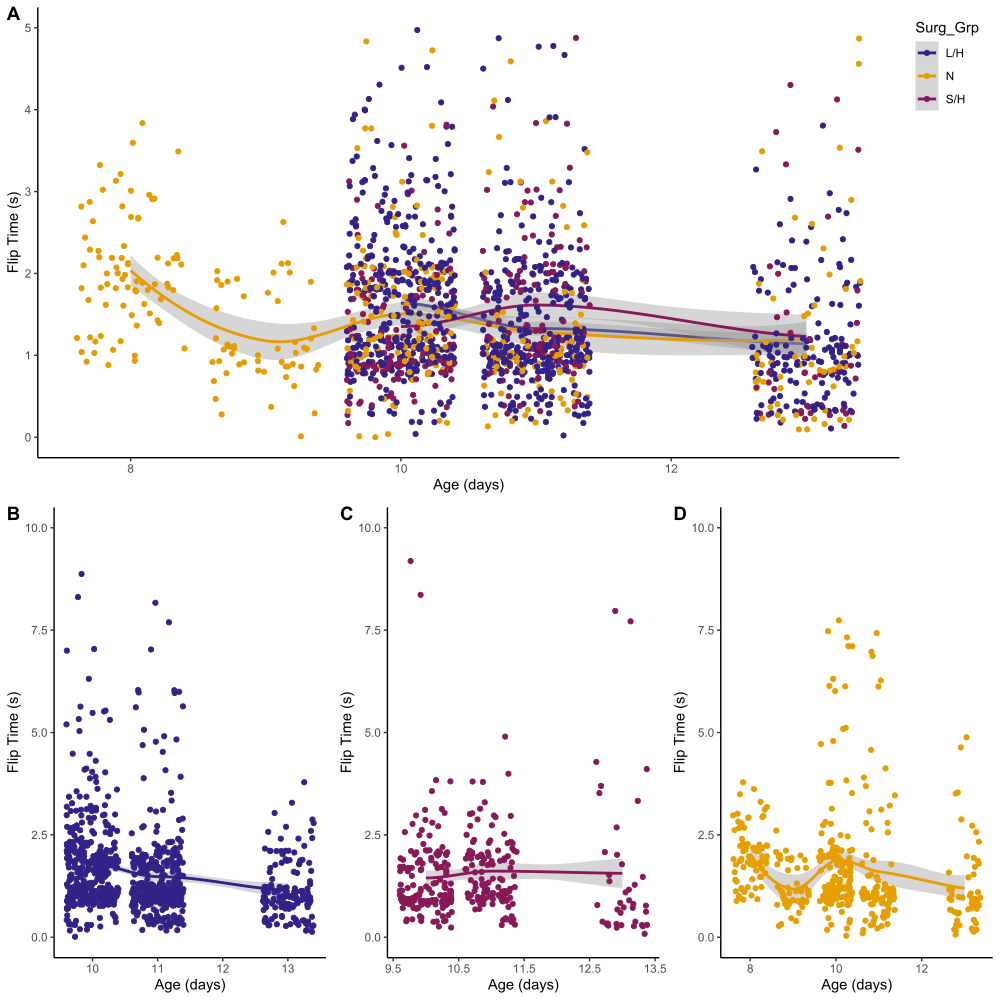

Supplement: Supplementary file 8 [file Image11.tiff]

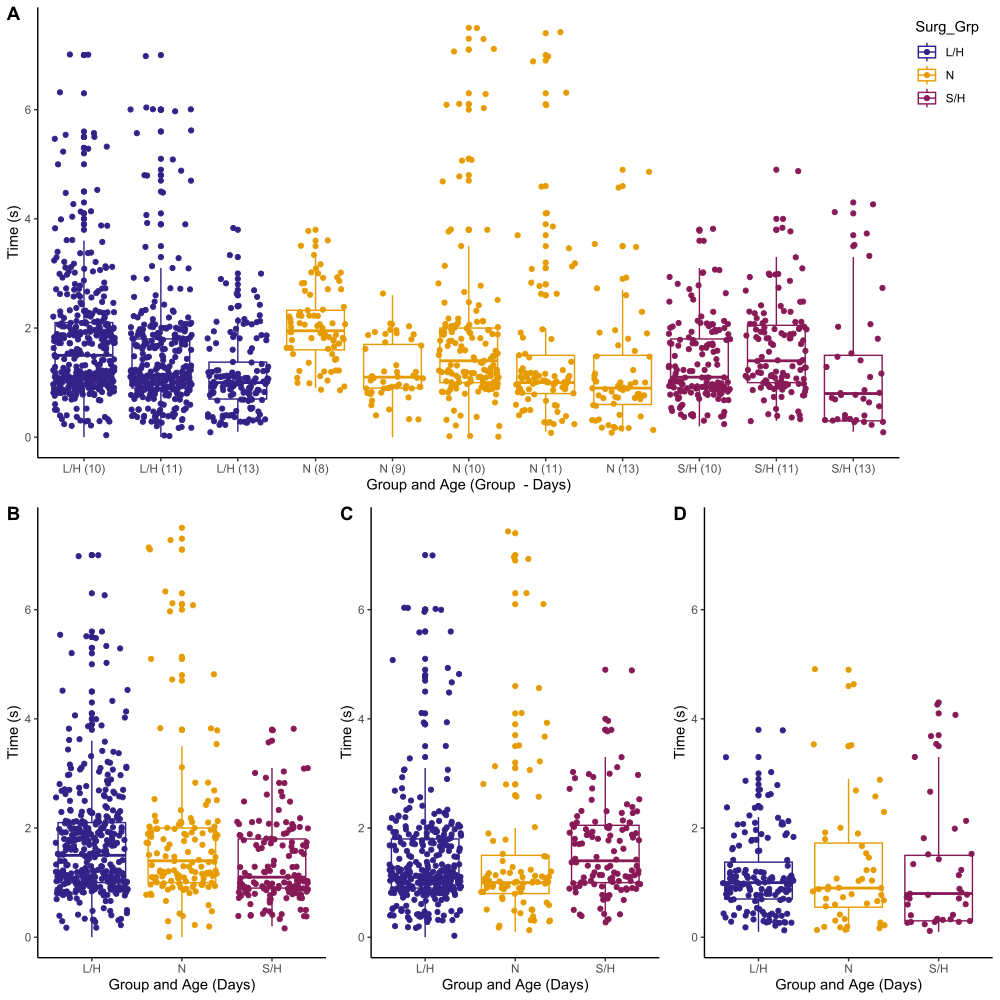

Supplement: Supplementary file 9 [file Image10.tiff]

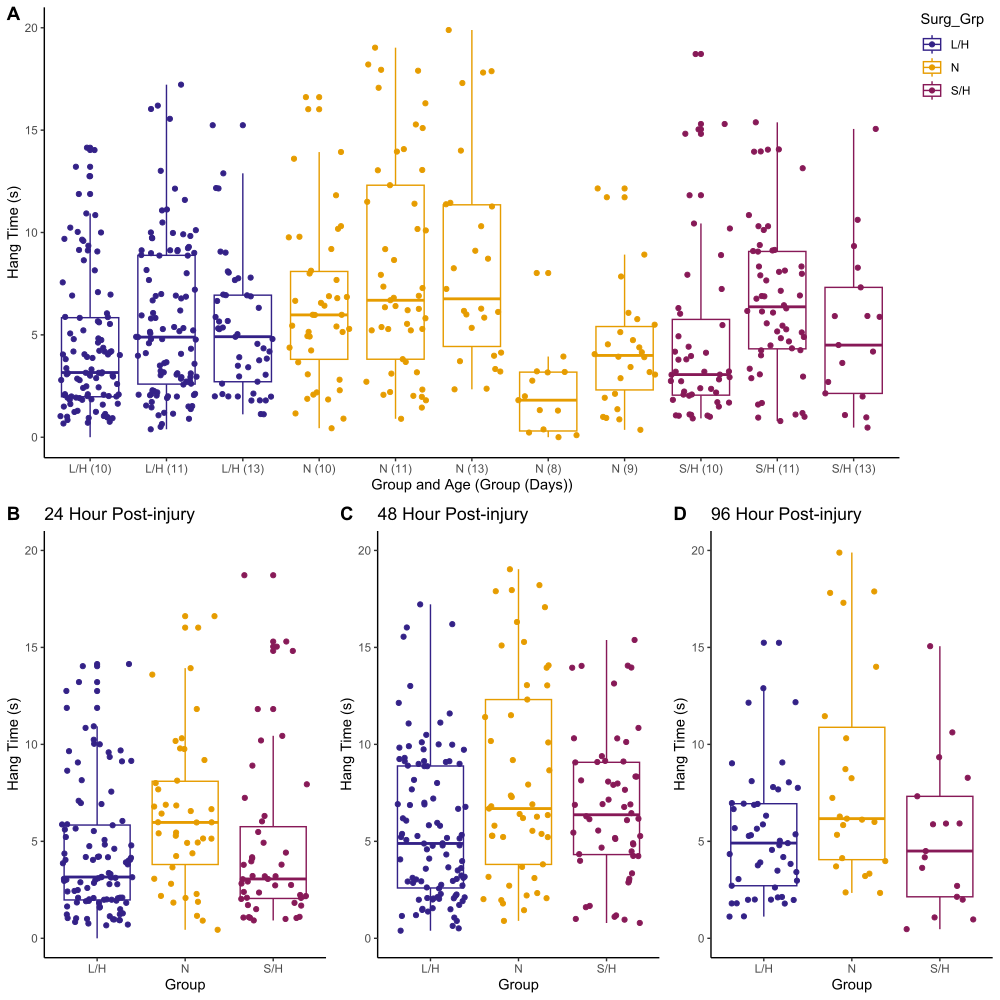

Supplement: Supplementary file 10 [file Image12.tiff]

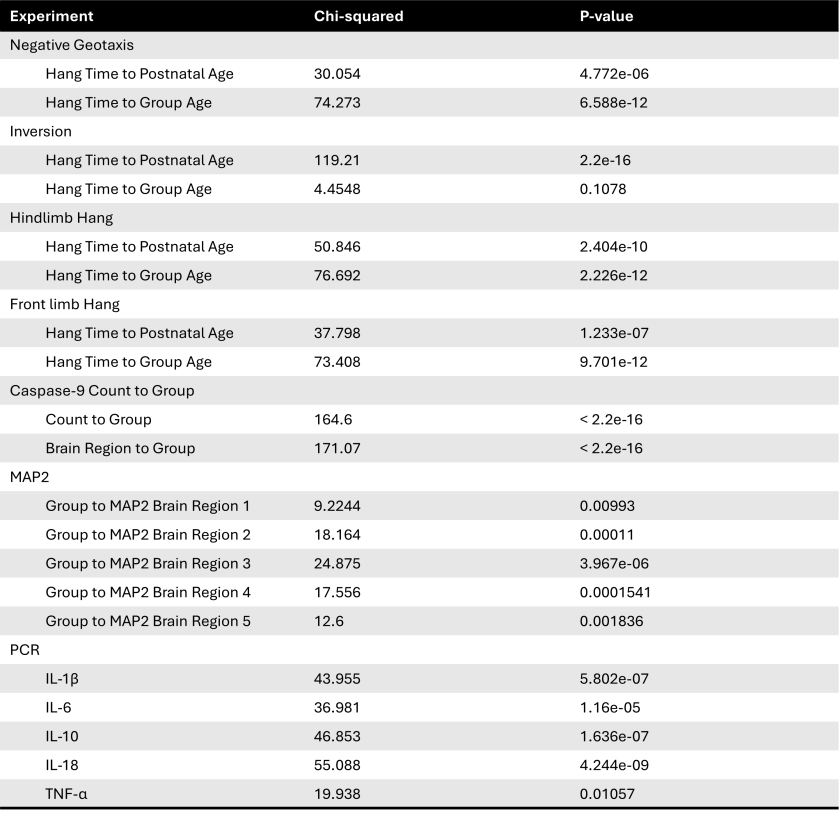

Supplement: Supplementary file 11 [file Image6.tiff]

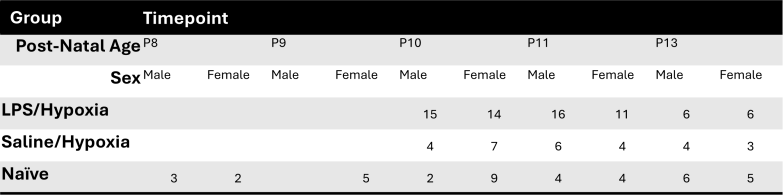

Supplement: Supplementary file 12 [file Image2.tiff]

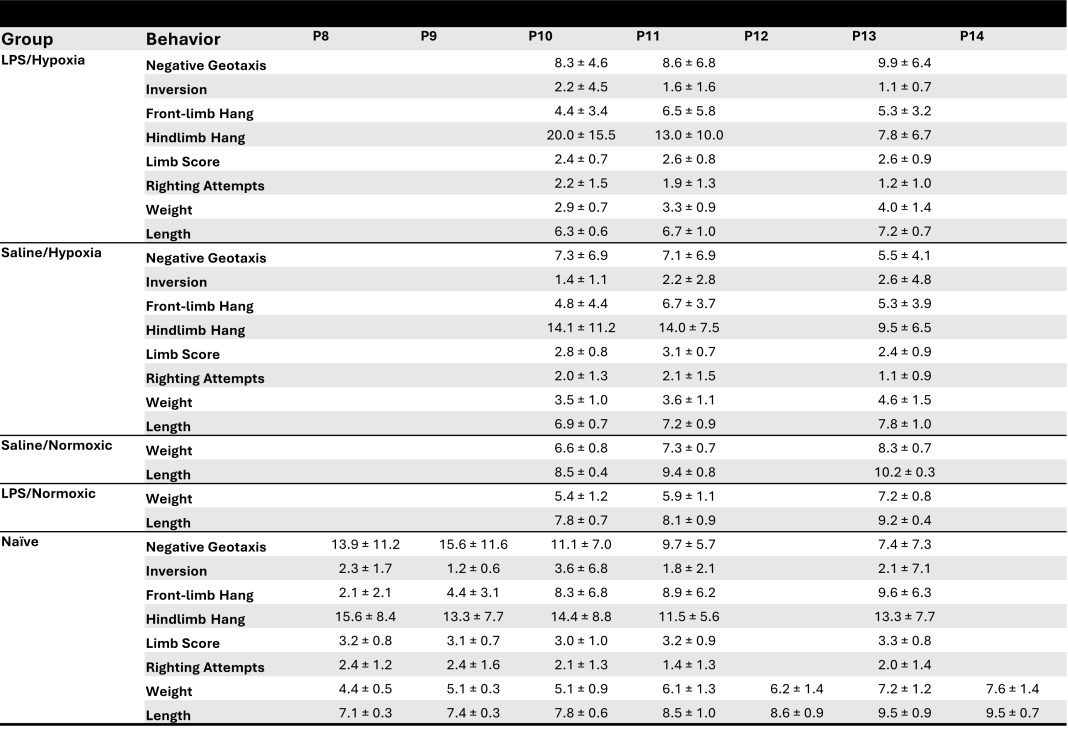

Supplement: Supplementary file 13 [file Image4.tiff]

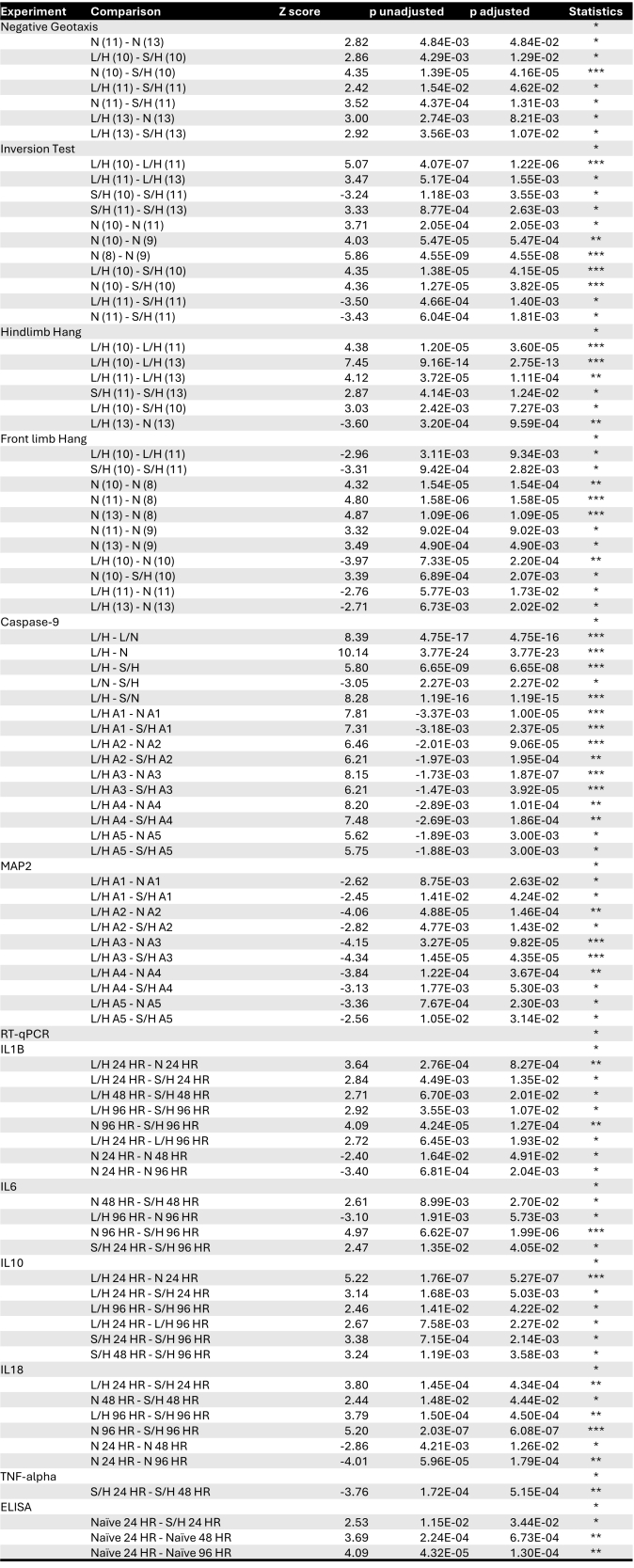

Supplement: Supplementary file 14 [file Image7.tiff]
