# Supplementary material for: Inflammation mediated brain damage and cytokine expression in a maternally derived murine model for preterm hypoxic-ischemic encephalopathy
Source: Front Syst Biol. 2025 Jul 1;5:1517712. doi: 10.3389/fsysb.2025.1517712 (PMC12342018; doi:10.3389/fsysb.2025.1517712)
Supplement: Supplementary file 7 [file DataSheet1.pdf]

Inflammation Mediated Brain Damage and Cytokine Expression In A Maternally Derived Murine Model For Preterm Hypoxic Ischemic Encephalopathy

Tyler C Hillman[1,3], Braeden Jacobson[3], Kiara Piaggio[4], Marlene Lopez[1], Nicholas Iwakoshi[1], and Christopher G Wilson[1,2,3]

1. Lawrence D. Longo, MD Center for Perinatal Biology

2. Department of Pediatrics, Loma Linda University Medical Center

3. Loma Linda University, School of Medicine

Loma Linda, CA 92350

4. Whittier College, Department of Biology, Whittier, CA 90602

Keywords: Hypoxia, Ischemia, Preterm, Neonate, Maternal Inflammation, Fetal Inflammation, Biomarkers

Corresponding Author:

Christopher G. Wilson, PhD

11223 Campus St

Loma Linda, CA 92350

Desk: 909-651-5895

Email: cgwilson@llu.edu

Supplemental Methods

Behavioral

Inversion

To test the animal’s ability to right themselves, we built a small platform with a padded top covered by autoclavable fabric. Two video cameras recorded the tests with two different vantage points. The cameras were set up so there was a vantage point above the animal and one parallel to the platform. Pups were gently held on their backs for three seconds and then released. The pups were recorded until they returned to an upright position with all four paws flat on top of the platform. Each pup was removed and tested for a total of four trials per animal. Once complete, the animals were returned before running further behavioral tests.

We positioned the camera at the top of the test area and the time that the researcher’s hands were no longer in contact with the pup, was recorded and listed as the start time. The end time was recorded as the frame when the animal was upright and all four limbs were in contact with the chamber floor. The direction of pronation was recorded in reference to the animal's perspective while supine (i.e. on its back) if the pup turned toward its right side, we considered this right pronation. Some animals were not able to invert and achieve pronation, and we scored these animals as 10.0 seconds, the total allowed length of the assessment time.

Negative Geotaxis

We constructed a ramp using a ¼-inch polyacrylamide sheet inside a 3D printed stand to hold the sheet at a 45-degree angle concerning the tabletop. The ramp is covered with autoclavable fabric and a red line is drawn on the center to indicate the starting position for the animal. We placed the pups face down on the ramp and gently held them for 5 seconds before releasing them. We recorded video from the bottom and top of the ramp. We recorded the views of each pup until they rotated 180 degrees from the original start position or crossed the red line.

Negative geotaxis was analyzed using the video footage and recording start and end times from edited videos. The start time for the test is determined as the frame when the researcher's hands are no longer in contact with the animal. This time is then subtracted from the end time, when the pup's head and two front paws could be seen oriented uphill, roughly parallel to the red line. The direction of rotation was recorded from the perspective of the animal. In some instances, the animal never gained a solid footing on the ramp and slid towards the tabletop. When pups attempted to rotate but rolled off the ramp after orienting perpendicular to the red line, we scored the trial as the max time (45 s).

Front-limb Hang

Using a one-liter beaker, a thin wire (24 AWG) was strung across the lip of the beaker and tensioned to allow the pups to grip and hang without bending or warping the wire. The bottom of the beaker was padded with surgical gauze to prevent injury when they released the wire and fell into the

beaker. A camera was placed facing the ventral side of the pup, level with the rim, to analyze how many times the pup could pull themselves up on the wire (pull-ups). An additional camera was placed at 90° allowing an additional vantage point to record the pups' behavior. We suspended pups on the wire until they were able to grip the wire and then released them. The video was recorded until the animal dropped below a gradation mark on the side of the large beaker (500 ml beaker, marker at 200 ml gradation). Each pup was assessed for three trials with between 30 minutes to an hour's rest between each trial. Animals were tested in series allowing the pups to recover as much as possible. Start time was determined by the frame the investigator released the pup, and the end time when the first part of the pup crossed the 200 ml gradation marker. Pull-ups were only quantified if the pup manage to elevate 50% of its head above the rim of the beaker. Two exceptions were noted: 1) when the animal escapes the container without falling to the bottom, and 2) when the animal fails to grab the wire. When the animal escapes, the escape is noted and the test is considered void and repeated.

Hindlimb Hang

Using a large 500 ml graduated cylinder, surgical gauze was placed in the bottom of the cylinder to provide padding for the pups to fall safely on. A gradation mark on the side of the cylinder recorded the stop time of the drop. Pups were placed so that both hindlimbs were on the lip of the graduated cylinder. We released the pups allowing them to hang from their hindlimbs. The time was recorded until the animals dropped below the line. All pups were assessed for three trials before returning them to the dam.

We calculated hang time by recording the total time the pup was able to suspend themselves. We set the end time when any part of the animal's body crossed a taped line at 400 ml (on a 500 ml graduated cylinder). We set the start time to the first frame where the researchers' fingers were visible being removed from the pup. Total elapsed time (start to end of trial) was recorded. Additionally, the number of attempts the animal took to right itself was recorded. This was visualized by straining the hindlimbs along with a rise in elevation of the animal's body. Finally, as was the case for forelimb hang, any time when the animal either escapes or fails to hold onto the lip of the cylinder is considered an invalid trial and repeated.

Visiopharm

Utilizing Visiopharm (Visiopharm, App 10016, version 2023.01.2.13695), the area was quantified utilizing apps thresholding with MAP2 set as channel F(1), lower threshold 11 and no upper threshold, Hematoxylin set as channel F(2) lower threshold 25 and upper threshold 190, finally Tissue gap or no stain set as channel F(2) lower threshold 190 and no upper threshold. Then the area of positive hematoxylin stain was divided by the total area minus the area of blank space included within the ROI for that tissue slice.
